# Biological Parts for *Kluyveromyces marxianus* Synthetic Biology

**DOI:** 10.3389/fbioe.2019.00097

**Published:** 2019-05-07

**Authors:** Arun S. Rajkumar, Javier A. Varela, Hannes Juergens, Jean-Marc G. Daran, John P. Morrissey

**Affiliations:** ^1^School of Microbiology, Centre for Synthetic Biology and Biotechnology, Environmental Research Institute, APC Microbiome Institute, University College Cork, Cork, Ireland; ^2^Department of Biotechnology, Delft University of Technology, Delft, Netherlands

**Keywords:** *Kluyveromyces*, synthetic biology, metabolic engineering, genome engineering, yeast

## Abstract

*Kluyveromyces marxianus* is a non-conventional yeast whose physiology and metabolism lends itself to diverse biotechnological applications. While the wild-type yeast is already in use for producing fragrances and fermented products, the lack of standardised tools for its genetic and metabolic engineering prevent it from being used as a next-generation cell factory for bio-based chemicals. In this paper, we bring together and characterise a set of native *K. marxianus* parts for the expression of multiple genes for metabolic engineering and synthetic biology. All parts are cloned and stored according to the MoClo/Yeast Tool Kit standard for quick sharing and rapid construction. Using available genomic and transcriptomic data, we have selected promoters and terminators to fine-tune constitutive and inducible gene expression. The collection includes a number of known centromeres and autonomously replication sequences (ARS). We also provide a number of chromosomal integration sites selected for efficiency or visible phenotypes for rapid screening. Finally, we provide a single-plasmid CRISPR/Cas9 platform for genome engineering and facilitated gene targeting, and rationally create auxotrophic strains to expand the common range of selection markers available to *K. marxianus*. The curated and characterised tools we have provided in this kit will serve as a base to efficiently build next-generation cell factories from this alternative yeast. Plasmids containing all parts are available at Addgene for public distribution.

## Introduction

Cell factories can serve as the basis of a new bio-based economy based on the sustainable production of fine chemicals, pharmaceuticals, nutraceuticals, and biofuels from engineered or native microbes. As of now, the most commonly-used eukaryotic cell factory is baker's yeast *Saccharomyces cerevisiae*. An ease of genetic manipulation, along with a wealth of genomic, genetic and biochemical knowledge, have made it able to produce a diverse range of compounds with *S. cerevisiae* from simple or economical feedstocks. However, non-*Saccharomyces* yeasts can provide several advantages over *S. cerevisiae* for building cell factories as they often possess desirable tolerances or metabolic traits that would otherwise need to be extensively engineered into baker's yeast (Wagner and Alper, 2016). In general, such yeasts have had niche applications in biotechnology but are being developed to be next-generation cell factories. *Kluyveromyces marxianus* is one such alternative yeast. While thermotolerant, fast-growing and able to use various carbon and nitrogen sources, it broadly has the same nutritional requirements and culture techniques as *S. cerevisiae*. Its natural strain diversity allows a wide number of phenotypes and gene variants to be exploited and combined to create an optimal cell factory chassis, and as a broadly Crabtree-negative species, it does not need to have metabolism routed away from ethanol production. While it has applications in the production of aroma compounds, fermented foods, and secreted enzymes (Morrissey et al., 2015; Gombert et al., 2016), *K. marxianus* is yet to be established as a metabolic engineering platform. Obstacles to such development include inefficient and random native gene targeting (hindering the stable expression of integrated heterologous genes), limited knowledge of its biochemistry and genetics, and a lack of standardised regulatory parts and expression systems on the level of baker's yeast. While such tools are starting to be developed, it still lacks the well-defined sets of regulatory elements to precisely control gene expression one uses in *S. cerevisiae*. As of now, individual parts are either selected from the native genome based on orthologues of genes of common parts from *S. cerevisiae* (Lee et al., 2013), or from other yeasts altogether (Chang et al., 2013), precluding the advantages of using *K. marxianus'* environmental triggers to fine-tune gene expression. Taken together, less than 20 native regulatory parts are currently in use for metabolic engineering (Bergkamp et al., 1993; Ball et al., 1999; Yang et al., 2015a; Gombert et al., 2016).

*Kluyveromyces marxianus*-specific techniques exist for the efficient *in vivo* assembly of large multigene constructs (Chang et al., 2012), and in conjunction with CRISPR/Cas9 (Löbs et al., 2017; Nambu-Nishida et al., 2017; Cernak et al., 2018) can allow us to specifically edit a genome, or efficiently target chromosmal integrations. Nonetheless *in vivo* assembly as it stands does not eliminate non-specific integrations of incomplete parts of the assembly. To further sidestep this problem, the MoClo standard, based on Golden Gate assembly, allows the efficient hierarchical *in vitro* assembly of multigene constructs either on episomal or integrative vectors for such purposes (Weber et al., 2011). It has been adapted for synthetic biology in diverse organisms, and is efficient enough to circumvent *in vivo* assembly. One variant of MoClo, the Yeast Toolkit (YTK), collects a number of well-characterised parts for *S. cerevisiae* (Lee et al., 2015). The YTK has 8 general classes of parts, defined by the 5' and 3' overhangs used for Golden Gate assembly, which allow directional cloning. Taken together, the parts of the original YTK and the system itself allow for the versatile construction of vectors for *S. cerevisiae* with several selection markers and integration sites of choice if needed.

**Figure 1:**
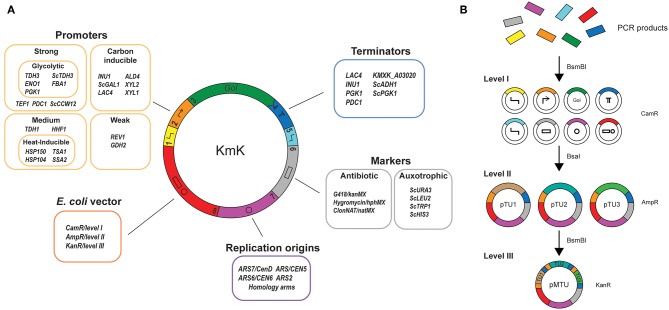
A collection of biological parts and synthetic biology tools for *Kluyveromyces marxianus*. **(A)** The Kluyveromyces Kit (KmK) provides parts according to the Yeast Toolkit (YTK) standard to express any gene of interest (GoI) under various conditions and expression platforms. Several constitutive and inducible promoters allow precise expression of a GoI under different conditions specific to *K. marxianus*, and terminator choice further fine-tunes gene expression. A number of metabolic and antibiotic markers can be used in wild-type yeast, or in auxotrophs generated by the CRISPR/Cas9 system provided. Finally, a number of species-specific origins and integration homology arms allow the expression of the GoI on a stable plasmid or as an integration cassette. **(B)** Hierarchy of YTK assemblies. Alternating the type IIS enzymes between assemblies allows construction of different plasmid “levels.” Starting from amplified PCR products, parts are cloned into level I plasmids for storage. At the time of cloning, they are given overhangs corresponding to the numbered parts used to build expression systems. From there, individual transcriptional units are built from level I plasmids either for use or for storage; these are level II plasmids. Finally, multiple TU-bearing level II plasmids can be combined to create multi-TU level III plasmids that are either episomal or integrative vectors. The use of different bacterial markers at each level allows us to use the previous level's plasmids directly for assembly.

The YTK's MoClo approach also sets up three tiers of plasmids for storage or use (Figure 1B). Level I plasmids correspond to part plasmids. A BsmBI site, and a BsaI site that after digestion will generate overhangs specific to that part type, flank each functional part. The BsmBI sites, in turn, are used to clone new parts into the entry vector YTK001 by Golden Gate assembly with that enzyme. At level II, level I plasmids are assembled together with BsaI to create gene expression cassettes or transcriptional units (TUs). Assembling TUs includes flanking them with synthetic and directional connector sequences which allow the construction of level III, plasmids. Here, multiple TUs are assembled together into a multigene expression or integrative vector, again with BsmBI based on unique overhangs present in the connectors.

The YTK, either with its standard or simply by using its parts collection, has since been used in diverse metabolic engineering and synthetic biology applications (Feng et al., 2015; Awan et al., 2017; Denby et al., 2018), and has already been adopted when cloning parts for *Pichia pastoris* (Obst et al., 2017). In this paper we present a collection of *K. marxianus*-specific parts and vectors, cloned to the YTK standard, for the genetic and metabolic engineering of this yeast. Over 30 constitutive and inducible promoters, terminators and centromeres and autonomously replicating sequence (ARS) elements have been selected from existing gene expression data and characterised as part of this collection. We have also identified a number of integration sites for the integration of single- and multi-gene constructs and have tested the best means to eliminate random integration. Taken together with existing YTK parts, our parts collection is a valuable set of tools for researchers working with *K. marxianus* as a cell factory.

## Materials and Methods

### Strains and Cultivation

*Kluyveromyces marxianus* strain CBS6556 (Westerdijk Fungal Biodiversity Institute, Utrecht, The Netherlands) was used for parts, unless specified otherwise. For part characterization and mutant creation, we used NBRC1777 [Biological Resource Centre, NITE (NBRC), Tokyo, Japan]. All strains used in this study are listed in Table 1. Yeast cultures were routinely grown in YPD broth (1% yeast extract, 2% peptone, 2% glucose) at 30°C. For promoter and terminator characterization, yeast was grown in synthetic drop-out medium without uracil [SD-ura; 0.17% yeast nitrogen base without amino acids or ammonium sulphate (Formedium, Hunstanton, UK), 0.5% ammonium sulphate and 0.19% SC-ura (Formedium)] containing 2% glucose or an alternate carbon source. For transformation of auxotrophic strains, yeast was grown in synthetic drop-out (SD) medium containing 2% glucose and lacking the appropriate nutrients (Formedium). For fermentation experiments, strains were cultured in minimal medium (MM) with 2% glucose (Fonseca et al., 2007). When needed, G418 or hygromycin (Fisher Scientific, Dublin, Ireland) was added to a concentration of 200 mg L^−1^ for selection or 150 mg L^−1^ for maintenance. Bacterial transformations used *E.coli* DH5α grown in LB medium (1% NaCl, 1% peptone, 0.5% yeast extract) supplemented with the appropriate antibiotics (100 mg L^−1^ ampicillin, 50 mg L^−1^ chloramphenicol or 50 mg L^−1^ kanamycin).

**Table 1:** List of *K. marxianus* strains used in this paper.

**Strain name**	**Source**	**Genotype**	**Comments**
CBS6556	Westerdijk Fungal Biodiversity Institute, Netherlands	Wild-type	
NBRC1777	NITE Biological Resource Center	Wild-type	
YBL001	This study	NBRC1777 *yku80-1*	Frameshift in codon 346 (double basepair deletion)
KmASR.005	This study	NBRC1777 *dnl4-1*	Frameshift in codon 11 (single base pair insertion)
YBL003	This study	NBRC1777 *nej1-1*	Frameshift in codon 61 (single base pair deletion)
KmASR.006	This study	NBRC1777 *ura3-1*	Frameshift in codon 139 (double base pair deletion)
KmASR.007	This study	NBRC1777 *his3-1*	Frameshift in codon 71 (single base pair insertion)
KmASR.008	This study	NBRC1777 *ura3-1 his3-2*	*HIS3:* Frameshift in codon 126 of *HIS3* (double base pair deletion)
KmASR.022	This study	NBRC1777 *leu2-1*	Frameshift across codons 124 and 125 double base pair deletion)
KmASR.023	This study	NBRC1777 *ura3-1 leu2-1*	*LEU2:* Frameshift across codons 124 and 125 (double base pair deletion)
KmASR.024	This study	NBRC1777 *his3-1 leu2-1*	*LEU2:* Frameshift across codons 124 and 125 (double base pair deletion)
KmASR.025	This study	NBRC1777 *ura3-1 his3-2 leu2-1*	*LEU2:* Frameshift across codons 124 and 125 (double base pair deletion)

### Selection and Amplification of Parts

Native promoters, terminators, and replicating sequences were amplified from the genomic DNA of *K. marxianus* CBS6556 (Jeong et al., 2012) unless specified otherwise. The sequences in question were identified from the genome sequence data (assembly GCA 000299195.2) following in-house gene prediction (Varela et al., 2017). In general, a promoter was defined as the first 1,000 bases upstream of a gene's start codon, unless this overlapped with a neighbouring gene or other features. Terminators were similarly defined as the first 250bp downstream of a gene's Stop codon (Curran et al., 2013). Parts were amplified from genomic DNA using Q5 High-Fidelity Polymerase (New England Biolabs, Ipswich, UK) and purified using a GeneJet PCR clean-up kit (Fisher Scientific) and eluted in sterile water prior to use. Yeast ARS elements and centromeres were selected from the literature, and either amplified from genomic DNA or from an appropriate plasmid. All assembled part plasmids with their Addgene plasmid IDs are listed in Table S1, and primers to amplify the parts are listed in Table S2.

### Golden Gate Assembly

Golden Gate assemblies were carried out as recommended (Lee et al., 2015) with minor modifications. In a typical reaction, 40 fmol of each insert (either as an existing level I plasmid or PCR product) were combined with 20 fmol of the plasmid containing the backbone for the final product to be assembled along with 1μL T4 DNA ligase 0.5 μL each of T7 DNA ligase (3,000U μL^−1^) and BsmBI or BsaI, (10U μL^−1^, NEB) and water to a total volume of 10 μL.

For Golden Gate assembly the protocol was as follows: 25 cycles of digestion and ligation (2 min at 42°C followed by 5 min at 16°C), followed by 10 min digestion at 60°C and 10 min inactivation at 80°C. For assemblies involving BsaI, the digestion step was carried out at 37°C for 3 min. When plasmids containing type IIS restriction sites that could not be removed, the final digestion and heat inactivation steps were eliminated.

Typically, half of a Golden Gate assembly was used to transform chemically competent *E.coli* DH5α. Transformants were screened by colony PCR with OneTaq Quick-Load DNA polymerase (NEB), using primers in the backbone vector flanking the insert if size permitted, or with one primer binding in the backbone and one in the insert. In the latter case, this was usually a primer used to amplify one of the component parts. For level I plasmids, colonies with the correct PCR product size had their plasmids extracted and inserts sequenced. For level II and III plasmids, PCR-positive colonies had their plasmids extracted and digested with NotI to verify the insert size All primers used to genotype strains are listed in Table S3, and maps of all the plasmids in Table S1 are included as Supplementary Material.

#### Construction of Reporter Plasmids

20fmol of a suitable “vector” level I plasmid from the Yeast Toolkit [pYTK083; (Lee et al., 2015)] were combined with 40fmol each of the following “insert” level I plasmids: left and right connectors from the Yeast Toolkit (pYTK002 and pYTK067), mVenus (pYTK034), a *kanMX* expression marker for G418 resistance, and the appropriate *K. marxianus* promoters (P1-19), terminators (T1-5), and centromere (typically C1; Table 2). A Golden Gate assembly for a level II plasmid was then carried out as described above. In total, 8 parts were assembled and transformed into *E.coli* per plasmid. Assembly of the construct was verified by colony PCR with ASR_K001F and ASR_K005R as primers and subsequent restriction mapping of plasmids from PCR-positive colonies with NotI. All original YTK plasmids used for assemblies are listed in Table S4.

**Table 2:** List of parts provided in the collection.

**Part**	**Source**	**YTK part type**	**Gene/systematic name/ description^**a**^**	**Addgene ID for plasmid containing part**
**REGULATORY PARTS**				
*PGK1pr/*P1	*K. marxianus* CBS6556	2/Promoter	3-Phospho-glycerate kinase/KMXK_0A03010	125034
*PDC1pr/*P2	*K. marxianus* CBS6556	2/Promoter	Pyruvate decarboxylase/KMXK_0F05000	125035
*ENO1pr/*P3	*K. marxianus* CBS6556	2/Promoter	Enolase/KMXK_0A03750	125036
*TDH1pr/*P4	*K. marxianus* CBS6556	2/Promoter	Glyceraldehyde-3-phosphate dehydrogenase isozyme 1/KMXK_0D02420	125037
*HSP150pr/*P5	*K. marxianus* CBS6556	2/Promoter	Hypothetical cell wall manno-protein HSP150/KMXK_0E02570	125038
*INU1pr/*P6	*K. marxianus* CBS6556	2/Promoter	Inulinase/KMXK_0A03230	125039
*TEF1pr/*P7	*K. marxianus* CBS6556	2/Promoter	Translation elongation factor EF alpha-1/KMXK_0G03180	125040
*REV1pr/*P8	*K. marxianus* CBS6556	2/Promoter	Deoxycytidyl transferase/KMXK_0F03560	125041
*ALD4pr/*P9	*K. marxianus* CBS6556	2/Promoter	Aldehyde dehydrogenase/KMXK_0E00190	125042
*GDH2pr/*P10	*K. marxianus* CBS6556	2/Promoter	Glutamate dehydrogenase/KMXK_0B07490	125043
*HHF1pr/*P11	*K. marxianus* CBS6556	2/Promoter	Histone H4/KMXK_0G01260	125044
*TSA1pr/*P12	*K. marxianus* CBS6556	2/Promoter	Thioredoxin peroxidase/KMXK_0D06030	125045
*HSP104pr/*P13	*K. marxianus* CBS6556	2/Promoter	Heat shock protein 104/KMXK_0D01210	125046
*SSA2pr/*P14	*K. marxianus* CBS6556	2/Promoter	Heat shock protein SSA2/KMXK_0B04700	125047
*TDH3pr/*P15	*K. marxianus* NBRC1777	2/Promoter	Glyceraldehyde-3-phosphate dehydrogenase isoform 3/KMAR_80062*^b^*	125048
*FBA1pr/*P16	*K. marxianus* CBS6556	2/Promoter	Fructose 1,6-bisphosphate aldolase/KMXK_0D04110	125049
*XYL1pr/*P17	*K. marxianus* CBS6556	2/Promoter	Xylose reductase/KMXK_0A01570	125050
*XYL2pr/*P18	*K. marxianus* CBS6556	2/Promoter	Xylitol dehydrogenase/KMXK_0H00420	125051
*LAC4pr/*P19	*K. marxianus* CBS397	2/Promoter	Beta-galactosidase*^c^*	125052
*INU1t/*T1	*K. marxianus* CBS6556	4/Terminator	Inulinase	125053
*LAC4t/*T2	*K. marxianus* CBS6556	4/Terminator	Beta-galactosidase	125054
*KMXK_A03020t/*T3	*K. marxianus* CBS6556	4/Terminator	KMXK_A03020t	125055
*PDC1t/*T4	*K. marxianus* CBS6556	4/Terminator	Pyruvate decarboxylase	125056
*PGK1t/*T5	*K. marxianus* CBS6556	4/Terminator	3 Phospho-glycerate kinase	125057
**ORIGINS OF REPLICATION**				
*KmARS7/CenD/*C1	p11256/*K. marxianus* DMKU3-1042 (Yarimizu et al., 2013; Varela et al., 2017)	7/Origin	Minimal ARS and centromeric sequence	125059
*KmARS/CEN5/*C2	*K. marxianus* CBS6556	7/Origin	ARS and centromere from chromosome V	125060
*KmARS/CEN6/*C3	*K. marxianus* CBS6556	7/Origin	ARS and centromere from chromosome VI	125061
*ARS2/*C4	*K. marxianus* CBS6556	7/Origin	Minimal ARS from chromosome II	125063
**HOMOLOGY ARMS FOR INSERTION VECTORS**				
I1L/I1R	*K. marxianus* CBS6556	1, 7	Chromosome I:10187.11936 (*LAC4)*	125030/125063
I2L/I2R	*K. marxianus* CBS6556	1, 7	Chromosme V: 23743.21744 (Crick Strand) (downstream of *KmBDH1* and *KmBDH2*)	125031/125064
I3L/I3R	*K. marxianus* CBS6556	1, 7	Chromosome IV:388650.390345 (between *KmSWF1* and *KmARO1*)	125032/125065
I4L/I4R	*K. marxianus* CBS6556	1, 7	Chromosome IV:240042.241741 (downstream of *KmHSP104*)	125033/125066
**OTHER PARTS**				
*ScTRP1*/M1	*S. cerevisiae* CEN.PK 113-7D	6/Marker	Phosphoribosyl anthranilate isomerase expression cassette from *S. cerevisiae*	125058

a Based on the CBS6556 genome annotations generated in Varela et al. (2017);

b Based on the NBRC annotations in (Inokuma et al., 2015);

c *Based on the genome sequence assembled in Ortiz-Merino et al. (2018)*.

#### Construction of Integrative Reporter Constructs

For the construction of integrative reporter vectors to evaluate insertion sites, a similar 8-part Golden Gate assembly was carried out as above, except that pYTK002 was replaced with plasmids containing 850–900 bp left homology arms targeting an insertion site (I1L-I5L), and C1 was replaced with plasmids containing 850–900 bp right homology arms targeting the same insertion site (I1R-I5R). For the experiments carried out here, for the purpose of evaluating integration sites, mVenus was always under the control of the *PDC1* promoter (P2) and the *INU1* terminator (T1). Following assembly and transformation, transformants were screened by colony PCR with primers ASR_K001F and ASR_P002R, followed by NotI restriction digestion as above.

### Promoter and Terminator Characterization

We constructed mVenus (YFP) reporter plasmids to characterise promoter and terminator strength. To minimise variations in expression due to copy number, we used centromeric plasmids with a *kanMX* cassette. While evaluating promoter strengths alone the reporter plasmids used the inulinase terminator in common (*INU1t*). In a similar manner, we used histone B promoter (*HHF1pr*) in common to regulate YFP expression when evaluating terminator strengths. Three hundred nanogram of reporter plasmids were transformed into *K. marxianus* by the LiOAc/PEG method (Gietz, 2014). After 48 h growth on selective medium, three transformant colonies were inoculated into 2 mL YPD with G418 and grown overnight at 30°C with 200 rpm agitation. The following day, the cultures were diluted 100-fold into 2 mL SD medium with 150mg L^−1^ G418 (approximately corresponding to a starting optical density of 0.1) and grown at 30°C for 24 h with 200 rpm shaking. For promoter inductions at high temperatures and xylose, overnight cultures were typically inoculated to a starting OD of 0.2–0.3 so that a comparable cell number would be present after 24 h growth. The cultures were then diluted 5 to 20-fold in identical SD medium on a 96-well microtitre plate, and YFP fluorescence measured on a Sirius HT platereader (MWG/BioTek, Winooski, USA) with excitation and emission set to 485 and 525 nm, respectively (bandwidth 20 nm). After correcting for the autofluorescence of wild-type *K. marxianus*, the signal was normalised to cell number by dividing by the OD at 600 nm. Differences between normalised YFP values under different conditions were tested for statistical significance by a paired t-test, with *p* < 0.05 taken to be significant.

For the characterization of integration sites, 2 μg of integrative plasmid containing an YFP expression cassette and *kanMX* marker was digested with SgsI/AscI (Fisher Scientific) and transformed into yeast. The amount corresponds to approximately 400 fmol of insertion cassette. G418-resistant colonies were screened for correct insertion at the intended locus by colony PCR and these alone were selected for YFP measurements. When the *LAC4* locus was targeted, transformation plates were replica-plated onto YPGal (2% galactose, 2% peptone, 1% yeast extract) containing 200 mg L^−1^ G418 and 40 mg L^−1^ X-Gal (Melford Laboratories, Ipswich, UK). The inability of disrupted *LAC4* to metabolise X-Gal—and not produce a blue dye—was made use of to pre-screen transformant colonies before genotyping them.

### Genome Editing Using CRISPR/Cas9

The cross-yeast CRISPR/Cas9 plasmid pUDP002 (Juergens et al., 2018) was modified to pUCC001 to allow easy cloning of new guide RNA (gRNA) targets by Golden Gate assembly. The original gRNA expression cassette of pUDP002 consisted of a target gRNA and structural element flanked by self-cleaving hammerhead and HDV ribozymes at the 5′ and 3′ ends respectively (Ng and Dean, 2017). This was modified to contain a BsaI cloning site between the hammerhead ribozyme and gRNA structural element (Vyas et al., 2015). The new cassette was assembled from long oligonucleotides (Integrated DNA Technologies, USA) by annealing them in a thermocycler. A plasmid backbone was then amplified from the original pUDP002 using primers pUDP002-F and pUDP002-R, and Gibson assembly was used to create pUCC001 from the two parts.

When using CRISPR/Cas9 to edit a site in the genome, a gRNA sequence targeting the gene of interest was first predicted using the sgRNA software (Xie et al., 2014). Complementary oligonucleotides comprising the gRNA sequence are designed with 5′ and 3′ overhangs (5′-CGTC-3′ and 5′-AAAC-3′,) on the sense and antisense oligonucleotides respectively, creating sticky ends to be ligated into pUCC001 cut by BsaI. One hundred pico mole oligos are phosphorylated with 1 μL of T4 polynucleotide kinase (10U μL^−1^, NEB) in a total volume of 10 μL and then denatured and annealed. Fifty femto mole of the annealed gRNA insert is then used with 100 ng of pUCC001 in a Golden Gate reaction with BsaI. Following transformation, the correct insertion of the gRNA was subsequently verified by PCR using the primer Bsa-R and the sense oligo containing the gRNA target, and sequencing of the plasmid. gRNA targets used in this study are listed in Table S5.

Three hundred nanograms of a gRNA expression plasmid are used in a typical genome engineering experiment. As *K. marxianus* predominantly repairs double stranded breaks by non-homologous end-joining (Daley et al., 2005), mutations are created around the gRNA target site without providing a repair fragment (Cernak et al., 2018). After 48–72 h growth following a transformation, hygromycin-resistant colonies are then screened for mutations at the targeted locus by colony PCR and sequencing (Jakociunas et al., 2015). If the intended mutations create an observable phenotype (e.g., an auxotrophy), the transformed plate is replica-plated to appropriate medium to pre-screen colonies based on the phenotype. Colonies with frameshift mutations are then grown overnight in YPD and then passaged twice to fresh YPD cultures to ensure loss of the gRNA plasmid before being preserved. When multiple genes were to be edited, sequential mutation was performed; following each mutation, the gRNA plasmid was cured and the mutation confirmed before proceeding with transformation of the next gRNA expression plasmid.

## Results

### A Collection of Parts and Assembly Pipeline for *Kluyveromyces marxianus* Using the Yeast Toolkit Standard

The biological parts collected and characterized here are intended to provide the same functions for *K. marxianus* in all the relevant categories of YTK parts (Figure 1A). A number of the parts have been described and identified elsewhere (Bergkamp et al., 1993; Yang et al., 2015a), but we also provide a more substantial number of characterised promoters, terminators and integration sites for expression cassettes. A number of high- and low-copy number origins have also been identified from *K. marxianus* genomes, or created from minimal elements. We have included three centromeric elements—one minimal (Yarimizu et al., 2013) and two genomic (Iborra and Ball, 1994; Ball et al., 1999)—and a minimal ARS element (Cernak et al., 2018) for the construction of expression vectors (Table 2). While the parts can be used to construct *K. marxianus* expression systems in general, they are optimally compatible with selection markers, bacterial vectors and synthetic connectors in the original YTK available from Addgene (#1000000061). They allow the *in vitro* construction of cloning and expression systems for *K. marxianus* with the same flexibility one can for *S. cerevisiae*. Golden Gate assembly is an established *in vitro* assembly technique, and with it we were able to assemble up to 8 part-containing plasmids into reporter plasmids to characterize our parts.

### A Set of Native Promoters to Fine-Tune Gene Expression

When selecting native yeast promoters to use in strain engineering, an important source is gene expression studies under conditions of interest. While a number of gene expression studies have been carried out on *K. marxianus* strains, few of them have focused on strains with the potential to be synthetic biology chassis, or on have instead focused on explicitly industrial conditions (Gao et al., 2015; Schabort et al., 2016; Diniz et al., 2017). To select promoters, we turned to two studies based on the strain DMKU3-1042, which also has the best quality publicly-available genome. One was TSS-seq transcriptome data published alongside its genome (Lertwattanasakul et al., 2015), as well as a small-scale gene expression study using fluorescent reporters of genes involved in carbon metabolism (Suzuki et al., 2015). The former gave us the opportunity to select not only promoters with distinct strengths, but also those induced by high temperature and xylose. Following this, the corresponding promoters were then identified from the CBS6556 genome. We excluded promoters with internal BsaI or BsmBI sites, as we lacked information on regulatory elements to justify removing the sites by mutagenesis.

**Figure 2:**
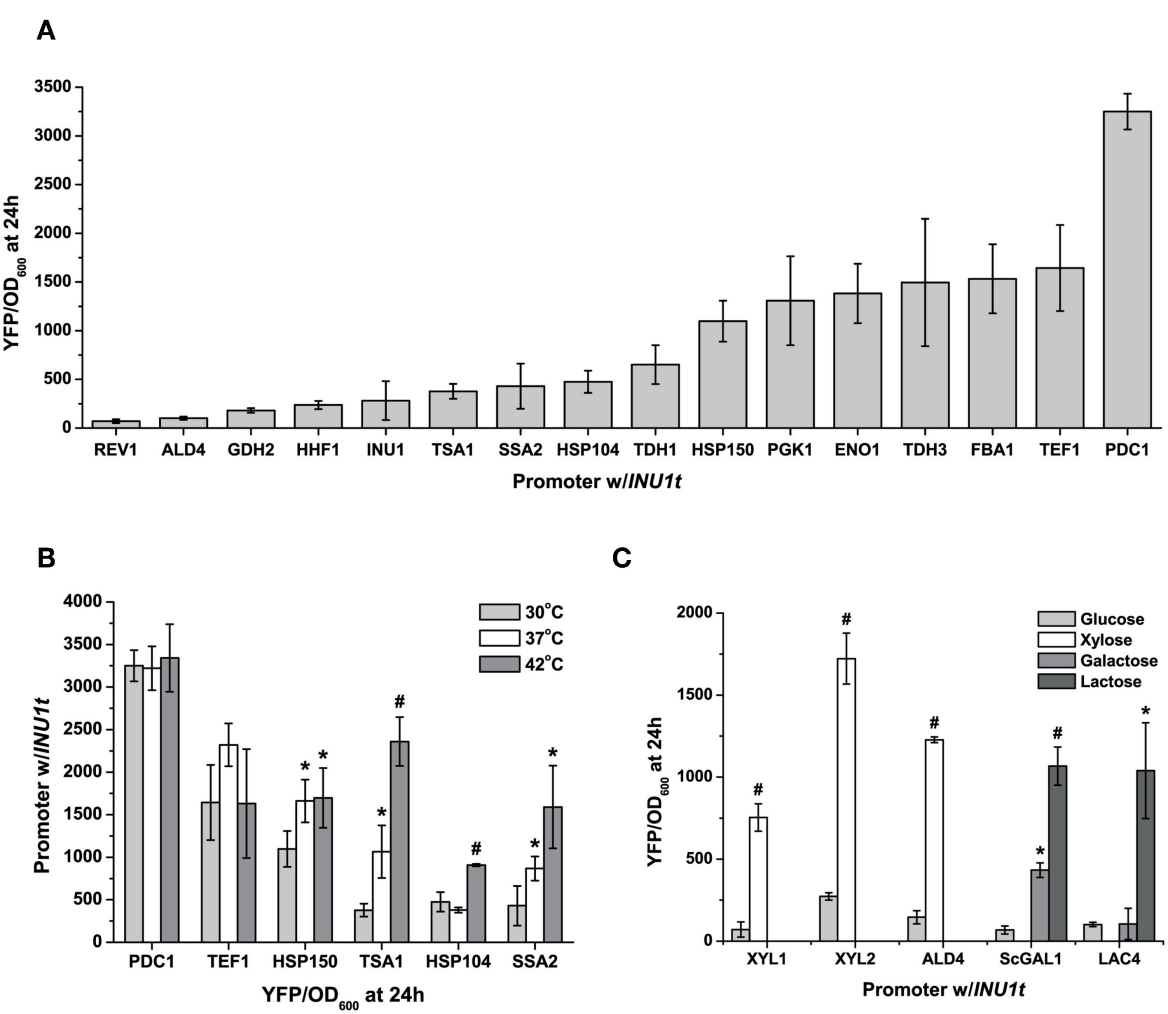
Characterization of *K. marxianus* promoters. **(A)** A set of constitutive promoters, taken from genes with diverse functions, provide a wide range of expression levels—nearly two orders of magnitude—as seen from the YFP output under standard conditions (24 h at 30°C, 2% glucose in synthetic complete medium) **(B)** Expression of heat-inducible promoters at three different temperatures at which NBRC1777 can grow. Constitutive promoters as well (*PDC1pr*) have a stable output at high temperatures. **(C)** The diverse carbon source utilization of *K. marxianus* gives us a unique induction signal for this yeast, as seen by the induction of promoters by lactose, galactose, and xylose. All data are normalised to cell number using OD, and are plotted as the mean ± s.d. of at least three replicates. YFP values significantly different from those under baseline conditions (30 degrees in drop-out medium with 2% glucose) are marked with an asterisk (*p* < 0.05) or a hash (*p* < 0.001).

This analysis allowed us to select 19 promoters: 10 constitutive and 9 inducible by heat, xylose, lactose and inulin, whose strengths we characterised using YFP reporter assays (Table 2, Figure 2A; Figure S1). While the promoter sequences came from CBS6556, we tested them in strain NBRC1777, due to its faster growth rate and superior thermotolerance compared to the former. Under standard conditions (30°C, glucose-rich medium), we provide a broad selection of promoter strengths for gene expression. From the weakest (*REV1pr*) to the strongest (*PDC1pr*) promoters, we can achieve a 40-fold range of gene expression in NBRC1777. The strongest promoters were those of genes involved in central carbon metabolism (*PDC1pr, FBA1pr, TDH3pr*), as well as that of the orthologue of the translation elongation factor EF-1α (*TEF1*) in *S. cerevisiae*. Interestingly, several of the same orthologous genes in *S. cerevisiae* also have strong promoters. However, the latter do not always achieve strong expression when used in *K. marxianus* (Figure S2)—a trade-off against the advantages of using an orthogonal yeast promoter.

*Kluyvermomyces marxianus's* thermotolerance provides a unique induction signal for this yeast's promoters. With this in mind, we picked three heat-inducible promoters (*HSP104pr, SSA2pr, TSA1pr*) with different fold-induction at high temperatures, and one intended to be stable at ambient and high temperatures (*HSP150pr*) based on expression data for the strain DMKU3-1042 as earlier (Lertwattanasakul et al., 2015). All of our inducible promoters selected from expression data exhibited induction under the relevant conditions, though not all to the same extent (Table S6). When tested at 37 and 42°C, *TSA1pr, SSA2pr*, and *HSP104pr* promoters induced to different levels. Expression of YFP by *HSP150pr*, as predicted, remained relatively stable at higher temperatures, inducing weakly when exposed to 37°C but no further at 42°C (Figure 2B). The other three promoters offer a range of induction varying from 2 to 6.5-fold depending on the temperature. *TSA1pr* has the strongest fold-induction at 42°C and the highest measured fluorescence at high-temperature, whereas that of *SSA2pr* remains at increasing medium-to-high levels (relative to the constitutive promoters) as temperature increases (Figure 2B). In comparison, two strong promoters at 30°C—*PDC1pr* and *TEF1pr*—had their YFP expression relatively unchanged by increased temperature.

Another feature of *K. marxianus's* physiology that makes it an attractive cell factory chassis is its ability to utilize a wider range of carbon sources than *S. cerevisiae*, thus circumventing the need to engineer this yeast to consume the carbon sources in question. Wild-type *K. marxianus* can consume sugars found in plant (xylose and inulin) and dairy (lactose) waste, thus allowing reduced costs if using these as feedstocks for fermentations. For xylose induction, we cloned and tested the induction of three xylose-inducible promoters: *XYL1pr, XYL2pr*, and *ALD4pr*. The xylose reductase *XYL1* reduces xylose to xylitol, this in turn converted to xylulose by the xylitol dehydrogenase *XYL2*. *XKS1* then phosphorylates it to xylulose-5-phosphate, which can then enter the non-oxidative branch of the pentose phosphate pathway. *ALD4pr* was chosen from a genome-wide expression study for its low background in glucose and high fold-induction by xylose (Lertwattanasakul et al., 2015). These promoters exhibited a 6-to-10-fold increase in YFP using xylose as a carbon source relative to glucose, with *XYL2pr* having the strongest fold-induction (Figure 2C). For a lactose-inducible promoter, we chose the promoter for the beta-galactosidase *LAC4*. Unlike the other promoters in this set, we chose *LAC4pr* from strain CBS397, known to grow well on lactose as a carbon source (Varela et al., 2017). It exhibited a 10-fold induction by lactose relative to glucose alone when used to express YFP in NBRC1777. In comparison, the *GAL1* promoter from *S. cerevisiae* was inducible by both galactose and lactose, but more strongly for the former (15-fold vs. 6-fold, Figure 2C).

### Terminators Provide a Second Level of Control Over Gene Expression

**Figure 3:**
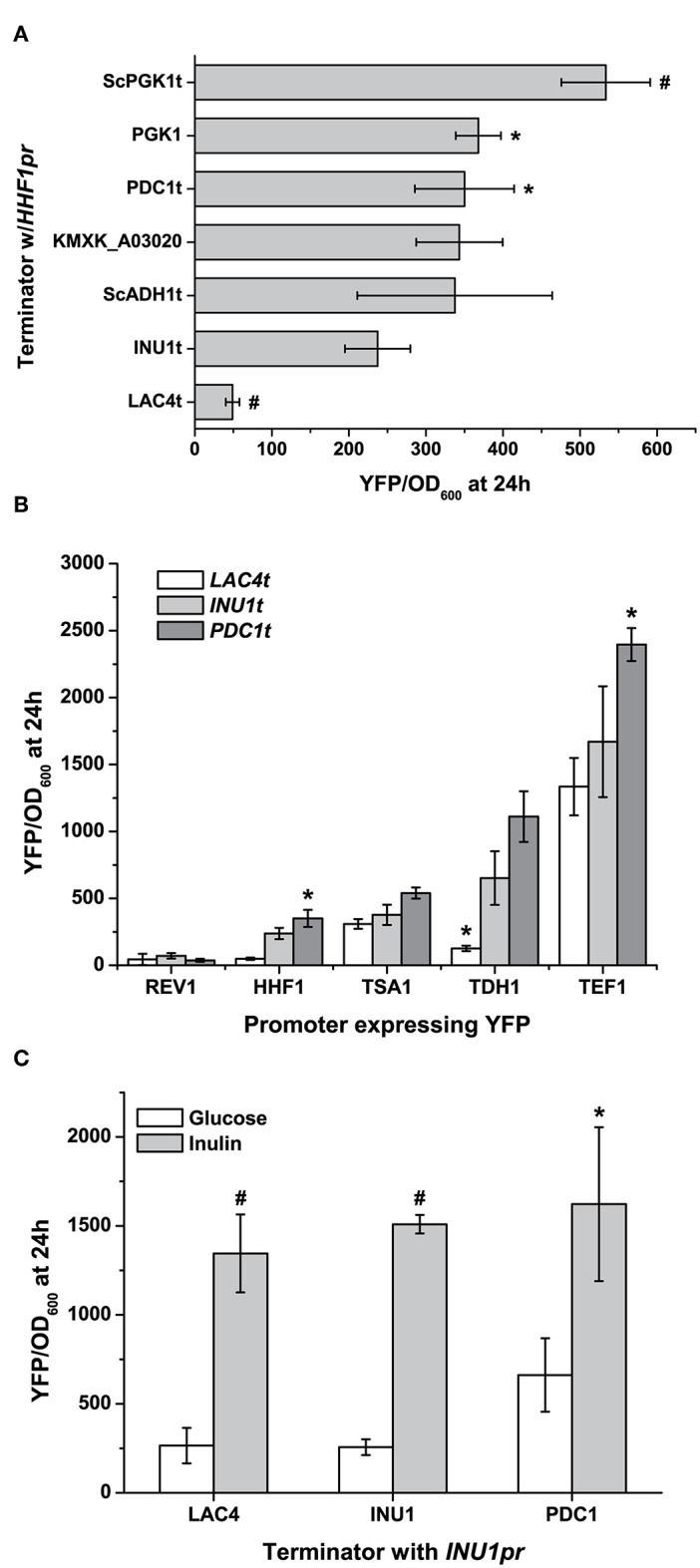
Using terminators to fine-tune gene expression. **(A)** Terminator choice can change gene expression by a factor of nearly 5 using native terminators, and this range can be even further exchanged if terminators from *S. cerevisiae* are used. **(B)** This can further be used to control gene expression which promoters and terminators are used combinatorially, though the starting strength of the promoter can influence the range. **(C)** Inducible promoters' expression is largely altered under non-inducing conditions when used to express YFP with different terminators. Here the strong induction of the inulinase (*INU1*) promoter by inulin is not significantly affected by terminator choice, while it can affect leaky expression under non-inducing conditions. All data are plotted as the mean ± s.d. of at least three replicates. YFP values significantly different from those under baseline conditions (expression using *INU1t*) are marked with an asterisk (*p* < 0.05) or a hash (*p* < 0.001).

Besides promoters, terminators can also control gene expression by affecting the lifetime of mRNA, and provide us with an extra means to fine-tune gene expression (Curran et al., 2013). While the terminators included with the original YTK aimed to keep gene expression output roughly constant (Lee et al., 2015), we have provided five native terminators to broaden the range of gene expression our parts can achieve (Figure 3A). Interestingly, two terminators from the YTK—those for *ScADH1* and *ScPGK1*—can change gene expression between them (as measured by YFP fluorescence) by nearly a factor of two in *K. marxianus*. The full ability of promoters to further optimise gene expression can be seen when a set of four terminators and three terminators are combinatorically used to express YFP. While the promoter of choice is still the dominant factor in determining the level of gene expression, choosing a “weak” or “strong” terminator can significantly affect expression as well (*TSA1pr* vs. *TDH1pr*, Figure 3B). In the case of inducible promoters, terminator effects are more pronounced under non-inducible conditions. Nonetheless, the increase in basal expression as seen in the inulinase promoter due to a change in a “stronger” terminator is enough to halve fold-induction by inulin (Figure 3C). In summary, depending on the promoter and the means of its induction, the terminators we provide could be used to minimise background, maintain a level of basal expression, or smooth out changes in expression during different conditions if needed when expressing a heterologous gene.

### Efficient CRISPR/Cas9 Editing With pUCC001

**Figure 4:**
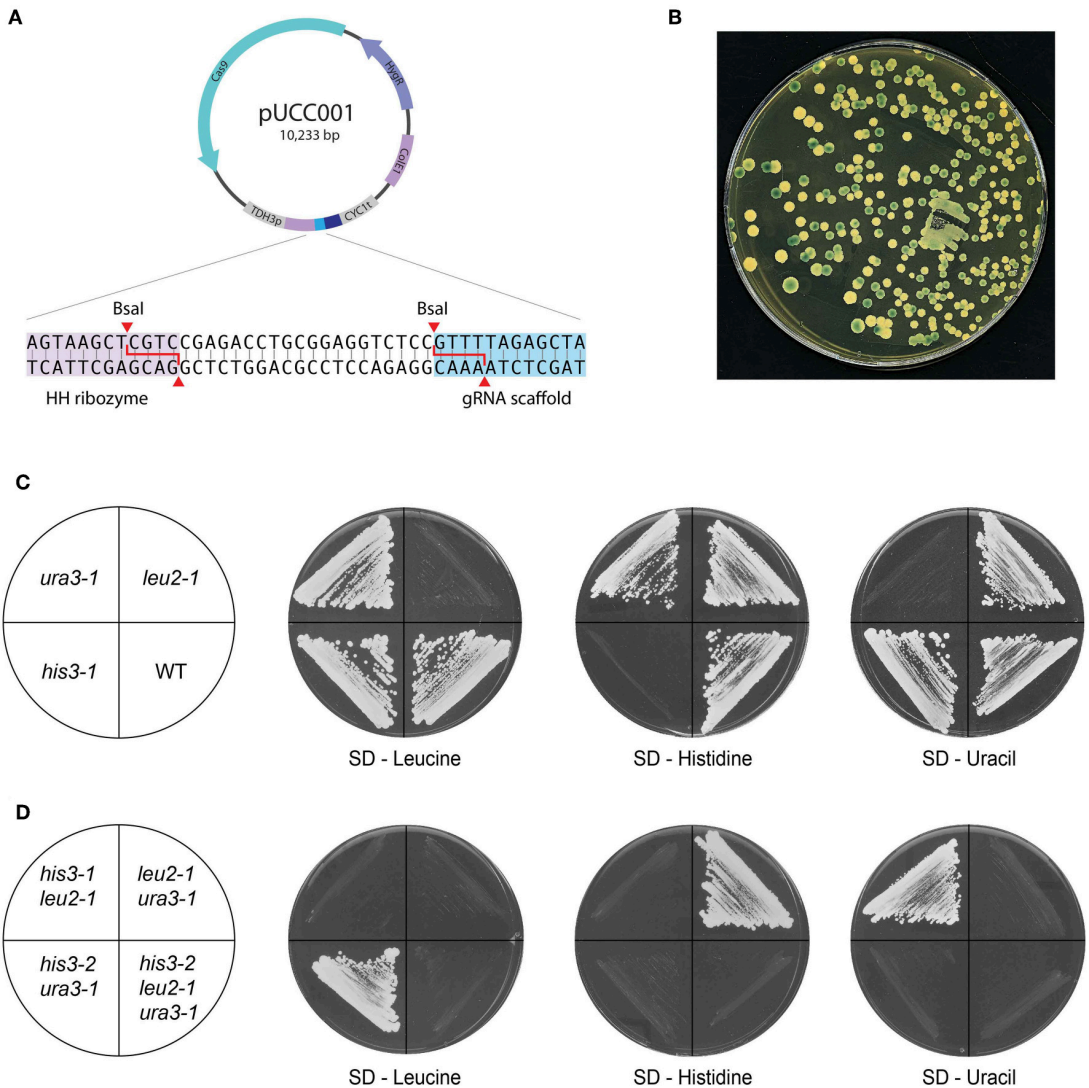
CRISPR/Cas9 genome editing with pUCC001. **(A)** Map of the plasmid. Based on pUDP002, the plasmid contains Cas9 expressed using the *A.adeninivorans* promoter, a pangenomic ARS and a gRNA expression cassette under the control of the *S. cerevisiae TDH3* promoter. The inset shows the cloning site inserted between the ribozyme units; two BsaI sites allow the construction of new gRNA targets using Golden Gate cloning. **(B)** Proof-of-principle of pUCC001 function by inactivation of the *LAC4* locus. Colonies grown YPGal + X-Gal, with a functioning copy of *LAC4* should produce a blue colour on cleaving X-Gal; here, over half of the colonies remain white due to CRISPR-mediated inactivation of *LAC4*. **(C,D)** Construction of single and multiple auxotrophs. With pUCC001, we were able to rapidly construct genome editing plasmids targeting orthologues of genes commonly inactivated in *S. cerevisiae* laboratory strains: *URA3, LEU2* and *HIS3*, both as **(C)** single and **(D)** multiple mutants. Frameshift mutations in each gene created the desired auxotrophies, and the strains so generated could be targeted with other plasmids to create defined double and triple mutants.

Our CRISPR/Cas9 editing plasmid pUCC001 takes advantage of the cross-species pUDP002 system and makes it a more flexible and economical tool by introducing a BsaI cloning site for new gRNA targets (Figure 4A). In this way, annealed oligos containing overhangs matching those generated by the BsaI sites in pUCC001 can be easily cloned into a cut plasmid, or using Golden Gate assembly. As a proof of principle, we transformed *K. marxianus* with pUCC001 containing a gRNA targeting the *LAC4* locus. *lac4* mutants are unable to convert X-Gal to a blue dye. We observed that over 50% of the hygromycin-resistant colonies did not turn blue when grown on medium containing galactose and X-Gal, as opposed to ~10–20% when a deletion cassette is used without CRISPR/Cas9 (Figure 4B, Table 3). As a demonstration of more practical applications, we used pUCC001 to rapidly generate defined single, double and triple auxotrophs for uracil, leucine, and histidine (Figures 4C,D; Table 1). Using gRNA plasmids targeting the *K. marxianus* orthologues of *URA3, HIS3*, and *LEU2* we created frameshift mutations in these genes leading to loss of function. Given that no repair fragment was used, the efficiency of mutations was good (≥50%); in general, we were able to retrieve the frameshift mutants in the figure by screening fewer than 8 colonies per transformation. The auxotrophic mutants so created allow us to use the orthogonal metabolic markers from the original YTK (*ScURA3, ScLEU2*, and *ScHIS3*), and show insignificant background when used in transformations with metabolic markers. Separately, the pUCC001 system was used to construct mutants in several other *K. marxianus* genes (Varela et al., 2019).

**Table 3:** Gene targeting and integration efficiencies at the *LAC4* locus in different backgrounds.

**Background/strain**	**G418—resistant colonies**	**White resistant colonies**	**Gene disruption efficiency, %^**a**^**	**Gene targeting efficiency, %^**b**^**
Wild-type/NBRC1777	863 ± 36	111 ± 13	12.9 ± 1.3	16.7 ± 5.9
*yku80-1/*YBL001	587 ± 71	587 ± 71	100	100
*dnl4-1*/KmASR.005	456 ± 44	455 ± 44	99.93	100
*nej1-1/*YBL003	469 ± 32	466 ± 32	99.36	100

*The data are presented as the mean ± s.d of three replicates*.

a The percentage of white colonies with respect to all blue and white colonies;

b *Colony PCR was performed on eight white colonies per replicate to check for correct integration of the YFP cassette at LAC4*.

### Inactivating Different Genes Involved in Non-homologous End-Joining Has Different Effects on Gene Targeting Efficiency

**Figure 5:**
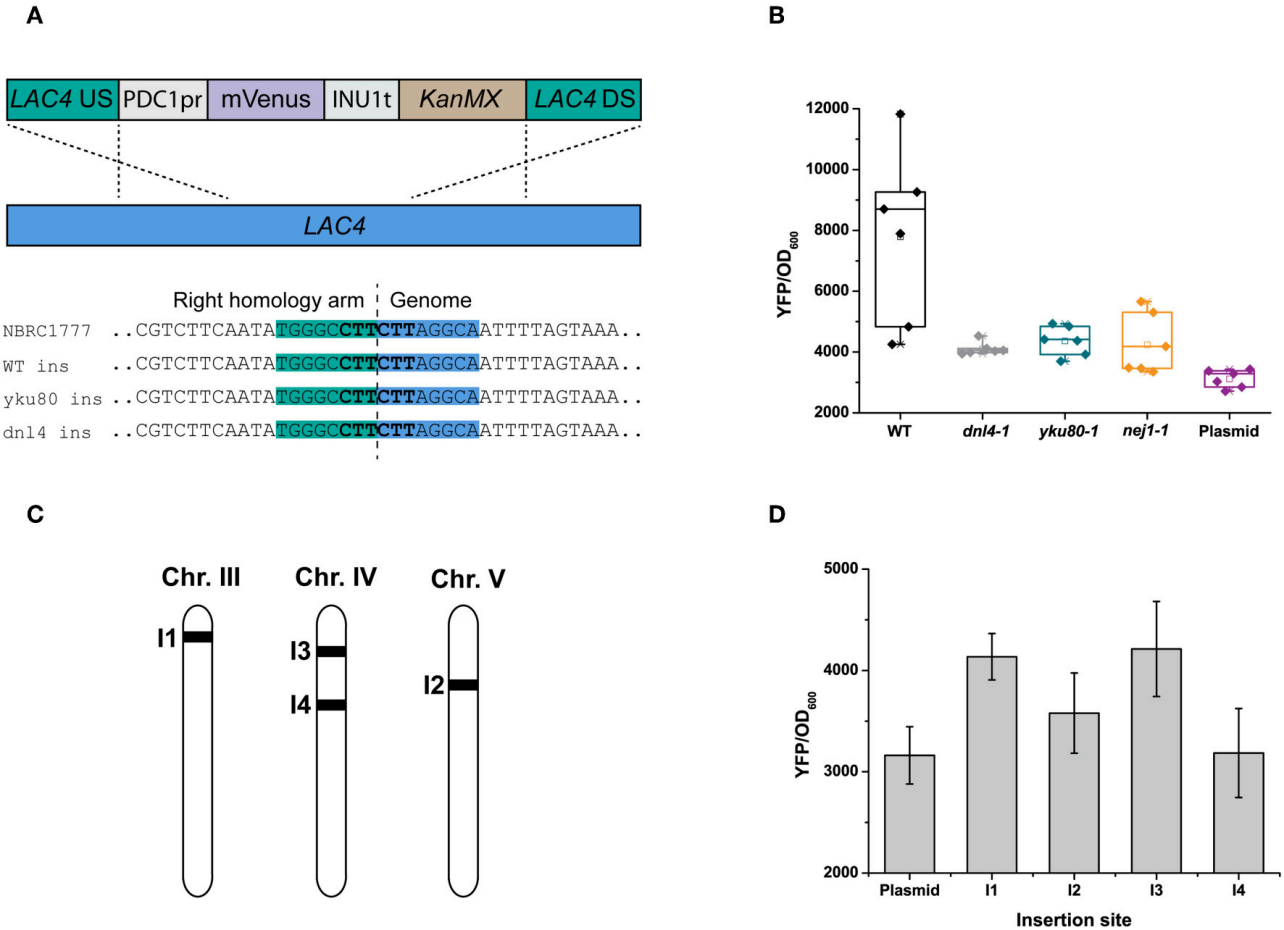
Random and targeted integration in *K. marxianus*. **(A)** Using homology arms ~880 bp in length, integration of a YFP reporter cassette (with marker) is seamless, with no sequence loss or modifications at the integration site irrespective of the genetic background (inset). **(B)** Inactivating *YKU80* or *DNL4* but not *NEJ1* is essential to eliminate random integrations. When targeting an YFP expression cassette at the *LAC4* locus, correct integration in a wild-type strain (as determined by genotyping) does not abolish random integration elsewhere, as seen by the spread in YFP fluorescence measured from five transformed colonies. YFP expression for the same cassette expressed on a centromeric plasmid is provided as a reference. **(C)** Genomic locations of insertion sites for gene expression cassettes, selected as described in the main text. **(D)** The effect of genetic context on expression levels of the same gene. The bar plots are plotted as the mean ± s.d. of at least three replicates.

While metabolically engineering strains it is advantageous to integrate heterologous gene cassettes for stronger and more stable expression. In yeasts other than *S. cerevisiae*, the ability to efficiently target gene integration is hindered by their usage of non-homologous end-joining (NHEJ) as the dominant DNA repair mechanism (Daley et al., 2005). As a result, random and incomplete integrations are frequent, and much larger targeting homology sequences are also required (up to 1 kb) compared to *S. cerevisiae* (50 bp) for a successful integration (Baudin et al., 1993; Choo et al., 2014). While random integrations of multiple copies of a gene can be advantageous in some biotechnology applications (Lin et al., 2017), it is equally important to integrate single copies of heterologous genes or pathways to rationally construct a cell factory or evaluate different metabolic engineering strategies. Inactivating any of the key genes involved in NHEJ—*YKU70/80, NEJ1* or *DNL4* (Abdel-Banat et al., 2010; Choo et al., 2014; Nambu-Nishida et al., 2017)—has been shown to increase targeted integration in *K. marxianus* by forcing it to use homologous recombination (HR) alone for DNA repair. It remains unclear if the different mutations suppress random integration to the same extent. To decide which NHEJ mutant gave us the highest rate of targeted integration, we tested the integration of a YFP expression cassette at the *LAC4* locus in backgrounds that were either wild-type or had either *YKU80, DNL4*, or *NEJ1* previously inactivated by CRISPR/Cas9 (YBL001, KmASR.005, and YBL003, respectively; Table 1). The cassette was flanked by 880 bp on either end targeting *LAC4* (Figure 5A). As with inactivation by CRISPR/Cas9, correct integration would disrupt the gene and render the yeast unable to break down X-Gal, allowing us to pre-screen colonies by blue/white selection for genotyping (Figure 4B). The YFP cassette would also allow us to determine if transformants contained multiple integrants in a semi-quantitative manner. Sequencing around the insertion site revealed that integration of the cassette was “seamless” in all backgrounds; the sequencing region immediately downstream of the insertion sites revealed no mutations surrounding the insertion point (Figure 5A).

Our experiments revealed that inactivating NHEJ resulted in disruption of the *LAC4* locus in nearly all transformants, as opposed to only occurring for about ~10% of transformants in wild-type *K. marxianus*. Furthermore, all white colonies of the NHEJ-deficient strains had the YFP cassette correctly integrated, as opposed to <20% for the wild-type strain (Table 3). In spite of correct integration, wild-type transformants still had a wide spread of measured fluorescent intensities (Figure 5B). This led us to hypothesise that correct integration in a wild-type strain does not preclude random integration. The contrasting low variation in YFP fluorescence seen in *DNL4* and *YKU80* mutants suggests that these backgrounds incorporate a single copy of the YFP cassette. On the other hand, *NEJ1* mutants seemed to incorporate multiple copies of YFP. In the case of the latter, it is admittedly unclear if these extra copies are integrated sequentially at *LAC4*, or elsewhere in the genome. In summary, it is clear that *DNL4* or *YKU80* are the best NHEJ components to inactivate to ensure targeted, single chromosomal integrations of DNA. Extrapolating from research in *S. cerevisiae*, our findings are given weight by the different roles in NHEJ of the proteins we targeted. *YKU80*, as part of the Ku complex, is the first protein to bind to and stabilise double-stranded breaks, followed by *DNL4* (Emerson and Bertuch, 2016). Therefore, the role of these “first responders” in DNA repair might make them better targets to thoroughly inactivate NHEJ. Furthermore, as other research has found that the binding of *DNL4* to DNA may not require *NEJ1* (Wu et al., 2008), and the latter enhances but is not essential for NHEJ entirely (Yang et al., 2015b), this may explain why the inactivation of *NEJ1* in YBL003 was insufficient to ensure single-copy gene integration. In general, expression for a single integrated cassette was higher than that when the same cassette was expressed on a low-copy-number plasmid using the same antibiotic marker (Figure 5B); the same trend was seen across several promoters (Figure S3).

### Evaluation of Sites for Chromosomal Integration

While selecting chromosomal integration sites in the *K. marxianus* genome, we considered three criteria: (i) providing a visible or easily detectable phenotype on integration, simplifying screening if necessary, (ii) near clusters of actively-transcribed genes, and (iii) near essential genes to ensure that only cells with correctly-inserted DNA would survive (Mikkelsen et al., 2012). For insertion sites of the first type, we chose the *LAC4* locus (I1); insertion there has a visible phenotype that does not affect core metabolism (as opposed to *ADE2*, for example). For selecting sites of the second type, we examined existing TSS-seq data (Lertwattanasakul et al., 2015) and found two such sites where the gene clusters were on the same coding strand: one each on chromosome IV and V (I2 and I4) (Figure 5C; Table 2). We also selected an insertion site upstream of *ARO1*, the pentafunctional protein involved in aromatic amino acid biosynthesis (I3); an incorrect insertion would result in auxotrophy for the aromatic amino acids phenylalanine, tyrosine, and tryptophan. While cloning the 5' (left) homology arms into level I plasmids, we added the overhangs matching the 5' multigene connector ConLS' by PCR, turning it into a type 1 part. This included the nested connector sequence containing the BsmBI site with the appropriate overhang for multigene constructs, which we added by PCR. This way the part with the homology arm can also be used to construct integrative vectors for a single expression cassette, as it still possesses the appropriate BsaI overhang at its 3' end to clone a TU in, as described below. The 3' (right) homology arms, as in the original YTK, contain BsaI overhangs corresponding to a yeast replicative origin, or type 7 part (Lee et al., 2015). Finally, the homology arms have AscI restriction sites directly flanking them to linearise the insertion vector prior to transformation, to improve integration efficiency.

Using these arms in place of connectors provided for TUs in the original YTK, we constructed novel integrative reporter vectors, each expressing YFP under the control of the strongest promoter we identified (*PDC1pr;* P2) and targeting I1 to I4. We then evaluated the integration efficiency at each site in KmASR.005, in each case using the same YFP expression cassette. Insertion efficiency was 100% at all loci, and while variations in YFP expression were observed at all loci, none varied by more than 25% (Figure 5D). However, this difference in expression may be more significant when expressing multiple genes in a heterologous biosynthetic pathway as opposed to a single fluorescent protein. Integrating the gene at I3, upstream of *ARO1*, did not cause the auxotrophies for Phe, Trp, and Tyr expected if the gene was disrupted, suggesting that the integration there was seamless as well. However, insertion sites near essential genes may not always be this accessible; another site we tested near an orthologue of the translation initiation factor *TIF1* yielded <10% correct integrations in the wild-type strain and no colonies in an NHEJ-deficient background (data not shown).

## Discussion

Standardised biological parts for the expression and maintenance of genes, and means to assemble them, are a cornerstone of synthetic biology to build new biological systems. On a more practical level, they allow us to accelerate the design-build-test cycle which forms the core of applied synthetic biology and its sister discipline, metabolic engineering. A versatile collection of these parts is the Yeast Toolkit collected 3 years ago. Grouped into eight categories of parts, the standard set forth by the YTK allows the hierarchical assembly of expression or storage vectors for expressing recombinant genes in *S. cerevisiae* with a wide range of selection markers, regulatory elements, and functional protein tags to fine-tune gene expression and engineer the genome as needed. Clearly, to move beyond being niche organisms in biotechnology, alternative yeasts require such part collections to make rapid metabolic engineering feasible. It is also advantageous to maintain a common standard for assembly to facilitate the exchange of parts between researchers and yeasts as needed. It was with this goal in mind that we selected and characterized the parts presented in this kit, while maintaining the YTK standard. Large part collections have been developed for other yeasts (Celinska et al., 2017; Prielhofer et al., 2017), and it is with such collections in mind that we have created ours. Using the established Golden Gate assembly protocols, we were able to assemble episomal and integrative reporter constructs from up to 8 component plasmids to characterize our parts.

Our extension of the YTK also includes the first collection of homology arms for insertion vectors targeting four loci in the *K. marxianus* genome, each with different characteristics. They are considered “full-length,” but can easily be shortened using PCR or re-cloning in the case of their use in NHEJ-deficient strains to an optimal length to minimize construct size without compromising gene targeting efficiency. As better genomic and transcriptomic knowledge of *K. marxianus* is acquired, more insertion sites and parts will be identified to be added to the modest set we provide. We also foresee the set being expanded by synthetic promoters, engineered promoters (as has been done with *INU1pr*) and secretion tags (Zhou et al., 2018).

While the promoters we have characterized are largely selected from strain CBS6556, all but one of them had >90% sequence similarity with strain DMKU3-1042 and with strain NBRC1777, where the characterization was carried out. Within the observed differences, only a few promoters do exhibit significant sequence differences that could affect gene expression between strains based on orthologous transcription factor binding sites from *S. cerevisiae* (Table S7). Sequence differences between promoters for the same gene in different yeast strains can have implications in gene expression, and therefore should be taken into consideration for experimental and industrial applications (Liu et al., 2015; de Paiva et al., 2018); however, at this stage not enough is known about *K. marxianus'* native transcription factors and regulatory network to functionally dissect our promoter sequences. Their measured activity under the selected expression conditions, and in two different strains, demonstrate their practical usability.

Alongside parts collections, the existence of genome editing tools for allelic replacement and deletion speeds up the creation of strains with defined mutant genotypes and mating, similar to standard *S. cerevisiae* lab strains. This also opens up the possibility of creating the best *K. marxianus* strains for the laboratory and industry using classical genetics methods and synthetic biology side-by-side (Cernak et al., 2018; Lee et al., 2018). It is with this end in mind that our collection also provides pUCC001, a Cas9/gRNA genome editing plasmid derived from the broad-host platform pUDP002 (Juergens et al., 2018), into which gRNA targets can be rapidly cloned by Golden Gate assembly simply as annealed and phosphorylated oligos. This saves the cost and time of cloning the entire gRNA expression cassette for every target as for the original plasmid. We have also cloned the *ScTRP1* expression cassette for use in future strains auxotrophic for tryptophan. The YTK standard makes it possible to multiplex gRNA expression using pUCC001. In theory, Cas9 and multiple gRNA cassettes could be separately cloned as level II plasmids and then reassembled into a level III “multi-TU” plasmid. The ease of assembly of both individual gRNA plasmids and the Golden Gate assembly would provide a credible, if not more efficient, alternative to existing multiplexing systems, (Löbs et al., 2018). However, further optimization of the gRNA expression system is necessary for an optimal *K. marxianus*—specific multiplexing system.

While investigating different NHEJ-deficient backgrounds, we found that inactivating *YKU80* or *DNL4*, but not *NEJ1*, was the best way to eliminate multiple or random integration. Identifying such a background is beneficial to improve the efficiency and specificity of *K. marxianus*-based *in vivo* assembly techniques such as PGASO (Chang et al., 2012), and to further define a genotype for a potential future “lab strain” for *K. marxianus*. As much as the versatility of the YTK standard is of relevance to synthetic biologists and metabolic engineers, the parts we have gathered may be of broader interest in the long term. *Kluyveromyces marxianus* is slowly emerging from its niche applications to become an alternative cell factory to *S. cerevisiae*. Several efforts have been, and are being made, to make it produce bio-based compounds of value (Cheon et al., 2014; Kim et al., 2014; Lin et al., 2017). The lack of standardised parts, and efficient synthetic biology tools and strategies has limited the scope or sophistication of these efforts. We believe this collection can enrich the existing synthetic biology landscape of *K. marxianus* and allow researchers to make more informed choices for the more efficient, predictable and practical design and testing of future cell factories for a bio-based economy.

## Author Contributions

AR and JV carried out the experimental work, interpreted the data and wrote the manuscript. HJ carried out the experimental work. J-MD and JM conceived the study, supervised the research, interpreted the data and contributed to writing the manuscript.

### Conflict of Interest Statement

The authors declare that the research was conducted in the absence of any commercial or financial relationships that could be construed as a potential conflict of interest.
