# Supplementary material for: Biological Parts for Kluyveromyces marxianus Synthetic Biology
Source: Front Bioeng Biotechnol. 2019 May 7;7:97. doi: 10.3389/fbioe.2019.00097 (PMC6515861; doi:10.3389/fbioe.2019.00097)
Supplement: Supplementary file 1 [file Table_1.docx]

Supplementary Material

Biological parts for *Kluyveromyces marxianus* synthetic biology

Arun S. Rajkumar^1^, Javier A. Varela^1^, Hannes Juergens^2^, Jean-Marc G. Daran^2^ and John P. Morrissey^1*^

^1^School of Microbiology / Centre for Synthetic Biology and Biotechnology / Environmental Research Institute / APC Microbiome Institute, University College Cork, Ireland

^2^Department of Biotechnology, Delft University of Technology, Van der Maasweg 9, 2629HZ Delft, The Netherlands

**Correspondence:**John P. Morrissey
J.Morrissey@ucc.edu

**Table S1**. Plasmids comprising the Kluyveromyces Kit.

| Plasmid name | YTK part type | Insert | E.coli marker | Addgene ID |
| --- | --- | --- | --- | --- |
| pUCC001 | n/a | SpCas9 and gRNA expression cassette | AmpR | 124451 |
| pKmK.I1L | 1 | Left homology arm, *LAC4* | CamR | 125030 |
| pKmK.I2L | 1 | Left homology arm, chromosome V: 23743..21744 | CamR | 125031 |
| pKmK.I3L | 1 | Left homology arm, *SWF1/ARO1* | CamR | 125032 |
| pKmK.I4L | 1 | Left homology arm, chromosome IV:240042..241741 | CamR | 125033 |
| pKmK.P1 | 2 | *PGK1pr* | CamR | 125034 |
| pKmK.P2 | 2 | *PDC1pr* | CamR | 125035 |
| pKmK.P3 | 2 | *ENO1pr* | CamR | 125036 |
| pKmK.P4 | 2 | *TDH1pr* | CamR | 125037 |
| pKmK.P5 | 2 | *HSP150pr* | CamR | 125038 |
| pKmK.P6 | 2 | *INU1pr* | CamR | 125039 |
| pKmK.P7 | 2 | *TEF1pr* | CamR | 125040 |
| pKmK.P8 | 2 | *REV1pr* | CamR | 125041 |
| pKmK.P9 | 2 | *ALD4pr* | CamR | 125042 |
| pKmK.P10 | 2 | *GDH2pr* | CamR | 125043 |
| pKmK.P11 | 2 | *HHF1pr* | CamR | 125044 |
| pKmK.P12 | 2 | *TSA1pr* | CamR | 125045 |
| pKmK.P13 | 2 | *HSP104pr* | CamR | 125046 |
| pKmK.P14 | 2 | *SSA2pr* | CamR | 125047 |
| pKmK.P15 | 2 | *TDH3pr* | CamR | 125048 |
| pKmK.P16 | 2 | *FBA1pr* | CamR | 125049 |
| pKmK.P17 | 2 | *XYL1pr* | CamR | 125050 |
| pKmK.P18 | 2 | *XYL2pr* | CamR | 125051 |
| pKmK.P19 | 2 | *LAC4pr* | CamR | 125052 |
| pKmK.T1 | 4 | *INU1t* | CamR | 125053 |
| pKmK.T2 | 4 | *LAC4t* | CamR | 125054 |
| pKmK.T3 | 4 | *KMXK_A3020t* | CamR | 125055 |
| pKmK.T4 | 4 | *PDC1t* | CamR | 125056 |
| pKmK.T5 | 4 | *PGK1t* | CamR | 125057 |
| pKmK.M1 | 6 | *ScTRP1* cassette | CamR | 125058 |
| pKmK.C1 | 7 | *KmARS7/CenD* | CamR | 125059 |
| pKmK.C2 | 7 | *KmARS/CEN5* | CamR | 125060 |
| pKmK.C3 | 7 | *KmARS/CEN6* | CamR | 125061 |
| pKmK.C4 | 7 | *KmARS2* | CamR | 125062 |
| pKmK.I1R | 7 | Right homology arm, *LAC4* | CamR | 125063 |
| pKmK.I2R | 7 | Right homology arm, chromosome V: 23743..21744 | CamR | 125064 |
| pKmK.I3R | 7 | Right homology arm, *SWF1/ARO1* | CamR | 125065 |
| pKmK.I4R | 7 | Right homology arm, chromosome IV:240042..241741 | CamR | 125066 |

**Table S2.** Primers used to amplify parts from genomic DNA. Overhangs for Golden Gate assembly are in boldface.

| **Primer name** | **Sequence (5’ to 3’)** | **Description** |
| --- | --- | --- |
| ASR_P001F | **GCATCGTCTCATCGGTCTCAAACG**CCAGTCACGTGTGAAACA | Forward primer for *PGK1* promoter |
| ASR_P001R | **ATGCCGTCTCAGGTCTCACATA**TTTGTATCTTTATATAGGTAGTGTGTA | Reverse primer for *PGK1* promoter |
| ASR_P002F | **GCATCGTCTCATCGGTCTCAAACG**TCCAGCGAATATACAGCGTG | Forward primer for *PDC1* promoter |
| ASR_P002R | **ATGCCGTCTCAGGTCTCACATA**GCAATTATTTGGTTTGGGTGT | Reverse primer for *PDC1* promoter |
| ASR_P003F | **GCATCGTCTCATCGGTCTCAAACG**AAGCCTTGAGCTTCGTGT | Forward primer for *ENO1* promoter |
| ASR_P003R | **ATGCCGTCTCAGGTCTCACATA**GTAGTTTGTGTTTGTTGTTGT | Reverse primer for *ENO1* promoter |
| ASR_P005F | **GCATCGTCTCATCGGTCTCAAACG**AACTACACTCCTATCGCAC | Forward primer for *TDH1* promoter |
| ASR_P005R | **ATGCCGTCTCAGGTCTCACATA**TTTTAAAATTATCTGAGTTGAGTTGTG | Reverse primer for *TDH1* promoter |
| ASR_P006F | **GCATCGTCTCATCGGTCTCAAACG**TCATTATTATTATTCATTATTCCACTTCTCG | Forward primer for *HSP150* promoter |
| ASR_P006R | **ATGCCGTCTCAGGTCTCACATA**GTATAAATCGGGGTATGTGTG | Reverse primer for *HSP150* promoter |
| ASR_P007F | **GCATCGTCTCATCGGTCTCAAACG**GAATTCTCAAACCGAAATGG | Forward primer for *INU1* promoter |
| ASR_P007R | T**ATGCCGTCTCAGGTCTCACATA**CTAACAAAAAAAAAATTAAATGTGTCACTTATG | Reverse primer for *INU1* promoter |
| ASR_P008F | **GCATCGTCTCATCGGTCTCAAACG**CAACGCATATGCTGCAAT | Forward primer for *TEF1* promoter |
| ASR_P008R | T**ATGCCGTCTCAGGTCTCACATA**TTTAATGTTACTTCTCTTGGAGTTAG | Reverse primer for *TEF1* promoter |
| ASR_P009F | **GCATCGTCTCATCGGTCTCAAACG**TGCTGGATGGAGAATTTTCG | Forward primer for *REV1* promoter |
| ASR_P009R | **ATGCCGTCTCAGGTCTCACATA**CTAGGTTGTCACTGACTAACTG | Reverse primer for *REV1* promoter |
| ASR_P010F | **GCATCGTCTCATCGGTCTCAAACG**TGTACAAATTATGGTATTAGGTAATG | Forward primer for *ALD4* promoter |
| ASR_P010R | **ATGCCGTCTCAGGTCTCACATA**GTTTTGTGCACGTGTGATT | Reverse primer for *ALD4* promoter |
| ASR_P011F | **GCATCGTCTCATCGGTCTCAAACG**TTTTGAACGCGAGGCTTTT | Forward primer for *GDH2* promoter |
| ASR_P011R | **ATGCCGTCTCAGGTCTCACATA**GTAGAATTTTTTTTTTGATATTATGATATTGTAC | Reverse primer for *GDH2* promoter |
| ASR_P012F | **GCATCGTCTCATCGGTCTCAAACG**GGTGATTCTTATATATGGTATACAATG | Forward primer for *HHF1* promoter |
| ASR_P012R | **ATGCCGTCTCAGGTCTCACATA**TTTATTTATTGATTGCTGTTTATTGC | Reverse primer for *HHF1* promoter |
| ASR_P013F | **GCATCGTCTCATCGGTCTCAAACG**CTAGCCTGGACCGGGATTT | Forward primer for *TSA1* promoter |
| ASR_P013R | **ATGCCGTCTCAGGTCTCACATA**TATTCCTTGTTAGCGGCAGC | Reverse primer for *TSA1* promoter |
| ASR_P014F | **GCATCGTCTCATCGGTCTCAAACG**TAGATTAATAAAATATATCTTGCGTTTCAA | Forward primer for *HSP104* promoter |
| ASR_P014R | **ATGCCGTCTCAGGTCTCACATA**TTTGTTCACTATATGGTTTGTATCTT | Reverse primer for *HSP104* promoter |
| ASR_P016F | **GCATCGTCTCATCGGTCTCAAACG**ACTATCTACACATTTTGTAACCA | Forward primer for *SSA2* promoter |
| ASR_P016R | **ATGCCGTCTCAGGTCTCACATA**TTTGCTTAATTTATTAATTGACTTTTTTG | Reverse primer for *SSA2* promoter |
| ASR_P019F | **GCATCGTCTCATCGGTCTCAAACG**GAAACTTGGGCCATGACA | Forward primer for *TDH3* promoter |
| ASR_P019R | **ATGCCGTCTCAGGTCTCACATA**GTGATGTGTAAAAGTGTGTGTGTA | Reverse primer for *TDH3* promoter |
| ASR_P020F | **GCATCGTCTCATCGGTCTCAAACG**GAGCGACAAACACACTCC | Forward primer for *FBA1* promoter |
| ASR_P020R | **ATGCCGTCTCAGGTCTCACATA**TTAGAATTTATTGGTTATTTGTGTGCTAAAAG | Reverse primer for *FBA1* promoter |
| ASR_P023F | **GCATCGTCTCATCGGTCTCAAACG**CCTGGGGAAACGGAAACC | Forward primer for *XYL2* promoter |
| ASR_P023R | **ATGCCGTCTCAGGTCTCACATA**GTTGATAATTTGTATTTTTGTTATTGGTAGCGC | Reverse primer for *XYL2* promoter |
| ASR_P024F | **GCATCGTCTCATCGGTCTCAAACG**GTTTCCAGTGTGTGATTCC | Forward primer for *XYL1* promoter |
| ASR_P024R | **ATGCCGTCTCAGGTCTCACATA**GTGTTTCTATACTGTGTTTTGGT | Reverse primer for *XYL1* promoter |
| ASR_P025F | **GCATCGTCTCATCGGTCTCAAACG**AGCCATCCAGGCCAGGTA | Forward primer for *LAC4* promoter |
| ASR_P025R | **ATGCCGTCTCAGGTCTCACATA**TCTTTCAGTTCTCGATGAGTATGTG | Reverse primer for *LAC4* promoter |
| ASR_T001F | **GCATCGTCTCATCGGTCTCAATCC**TCTGATCTGCTTACTTTACTAAC | Forward primer for *INU1* terminator |
| ASR_T001R | **ATGCCGTCTCAGGTCTCACAGC**CACCTTTCTAAGGTTAGAATAGAAAT | Reverse primer for *INU1* terminator |
| ASR_T004F | **GCATCGTCTCATCGGTCTCAATCC**GAATTTTATACTTAGATAAGTATGTACTTA | Forward primer for *LAC4* terminator |
| ASR_T004R | **ATGCCGTCTCAGGTCTCACAGC**CGAAAAAGTTGTCGGAATG | Reverse primer for *LAC4* terminator |
| ASR_T005F | **GCATCGTCTCATCGGTCTCAATCC**ATATAAAAACCGGTCACGTG | Forward primer for KMXK_A03020 terminator |
| ASR_T005R | **ATGCCGTCTCAGGTCTCACAGC**GTTGTTGTTAGCTAAGCTGT | Reverse primer for KMXK_A03020 terminator |
| ASR_T006F | **GCATCGTCTCATCGGTCTCAATCC**AGAGGGAGAGGATAAAGAGAT | Forward primer for *PDC1* terminator |
| ASR_T006R | **ATGCCGTCTCAGGTCTCACAGC**TGGTAACTTGTAACTTGTAACTTG | Reverse primer for *PDC1* terminator |
| ASR_T007F | **GCATCGTCTCATCGGTCTCAATCC**GTGCATCATTGACCTTTCTAA | Forward primer for *PGK1* terminator |
| ASR_T007R | **ATGCCGTCTCAGGTCTCACAGC**TGTGTATTTTTCGTATTTTAATTAATATTTATG | Reverse primer for *PGK1* terminator |
| ASR_M002F | **GCATCGTCTCATCGGTCTCATACA**CAATCAGTAAAAATCAACGGTTAAC | Forward primer for *ScTRP1* cassette |
| ASR_M002R | **ATGCCGTCTCAGGTCTCAACTC**CATTGCGGTGAAATGGTAAA | Reverse primer for *ScTRP1* cassette |
| ASR_C002F | **GCATCGTCTCATCGGTCTCAGAGT**GAGCTCCTTTCATTTCTGAT | Forward primer for *KmARS/CEN5* |
| ASR_C002R | **ATGCCGTCTCAGGTCTCATCGG**GGATCAATTGAAGTTTTATCCAA | Reverse primer for *KmARS/CEN5* |
| ASR_C003F | **GCATCGTCTCATCGGTCTCAGAGT**TGATCCAAGTCTGAAGGT | Forward primer for *KmARS/CEN6* |
| ASR_C003R | **ATGCCGTCTCAGGTCTCATCGG**TTCAGTCTTAGTTCACCTTAAACA | Reverse primer for *KmARS/CEN6* |
| ASR_C004F | **GCATCGTCTCATCGGTCTCAGAGT**CAATACTTTCTAACATTGACCATTTTG | Forward primer for *ARS2* |
| ASR_C004R | **ATGCCGTCTCAGGTCTCATCGG**GTCCCAGGTCTCTATAGTG | Reverse primer for *ARS2* |
| ASR_I001LF_MTU | **GCATCGTCTCATCGGTCTCACCCTTTCAGGCGCGCCG**AAAGTGATTGAAGAACCCT | Forward primer for I1L |
| ASR_I001LR_MTU | **ATGCAGGTCTCACGTTCGTCTCATCAGTCTAGATGCGAATTC**TTAAGCAATTGGATCCTACC | Reverse primer for I1L |
| ASR_I001RF | **GCATCGTCTCATCGGTCTCAGAGT**TGCTTAATTAGCTTGTACATGG | Forward primer for I1L |
| ASR_I001RR | **ATGCCGTCTCAGGTCTCATCGG**CTGAGGCGCGCCGAAGGCCCATATTGAAGAC | Reverse primer for I1L |
| ASR_I002LF_MTU | **GCATCGTCTCATCGGTCTCACCCTTTCAGGCGCGCC**ACCGAATATTAAATAATCTTTTTTTATTTCTTAT | Forward primer for I2L |
| ASR_I002LR_MTU | **ATGCAGGTCTCACGTTCGTCTCATCAGTCTAGATGCGAATTC**ATCTCATCCTCTAATGACGTAAA | Reverse primer for I2L |
| ASR_I002RF | **GCATCGTCTCATCGGTCTCAGAGT**TGAAGCATAGAAGAAATGTTTAACCA | Forward primer for I2R |
| ASR_I002RR | **ATGCCGTCTCAGGTCTCATCGGCTGAGGCGCGCC**GGCATTTAATAAGAATTTGTCTTCAGA | Reverse primer for I2R |
| ASR_I003LF_MTU | **GCATCGTCTCATCGGTCTCACCCTTTCAGGCGCGCC**TCATACCGTTTATGATTGCATTTAC | Forward primer for I3L |
| ASR_I003LR_MTU | **ATGCAGGTCTCACGTTCGTCTCATCAGTCTAGATGCGAATTC**CGGTTAGGAATATTAGCTAGATTG | Reverse primer for I3L |
| ASR_I003RF | **GCATCGTCTCATCGGTCTCAGAGT**AATTCAAGGTTGTTCAATATTCCGAT | Forward primer for I3R |
| ASR_I003RR | **ATGCCGTCTCAGGTCTCATCGGCTGAGGCGCG**CCAACACAAATTGTGGTTGCCA | Reverse primer for I3R |
| ASR_I004LF_MTU | **GCATCGTCTCATCGGTCTCACCCTTTCAGGCGCGCCG**GTGTTGCATTAGTATCGC | Forward primer for I4L |
| ASR_I004LR_MTU | **ATGCAGGTCTCACGTTCGTCTCATCAGTCTAGATGCGAATTC**TGTTCATAAGAACATGGTTTATGGA | Reverse primer for I4L |
| ASR_I004RF | **GCATCGTCTCATCGGTCTCAGAGT**TTCACAATGGAGGGAGTG | Forward primer for I4R |
| ASR_I004RR | **ATGCCGTCTCAGGTCTCATCGGCTGAGGCGCG**CCAAGTTGCCGGACACATATTAC | Reverse primer for I4R |

**Table S3.** Primers used for plasmid construction, genotyping and sequence verification. Overhangs are in boldface.

| **Primer name** | **Sequence (5’ to 3’)** | **Description** |
| --- | --- | --- |
| ASR_K001F | TTTGCTGGCCTTTTGCTC | General colony PCR forward primer, priming at the 3' end of ColE1 in all of YTK backbones |
| ASR_K002R | CATCTGGATTTGTTCAGAACG | Level I plasmid colony PCR reverse primer, priming at the 3' end of CamR in YTK001 |
| ASR_K004F | GCGATCACAGACATTAAC | Level II plasmid colony PCR reverse primer, priming in ConLS |
| ASR_K001R | ATTGGTAACTGTCAGACCAAGTTTA | Level II plasmid colony PCR reverse primer, priming at the 5' end of AmpR in YTK083 |
| ASR_K005R | GTCATCCGAGCGTGTATTG | Alternate level II plasmid colony PCR reverse primer, priming in ConR1 |
| ASR_K007R | CAGTCATCGGTATGATCTG | Alternate level II plasmid colony PCR reverse primer, priming in ConRE |
| ASR_K011F | AGAAAGTAATATCATGCGTCAA | Primes at 3’ end of *kanMX* cassette, used for confirming integrations |
| ASR_I1_US_F | CATTAGAACCTTTTTCAACACTC | Forward primer for verifying integrations at I1; primes 92bp upstream of I1L |
| ASR_I1_DS_R | CTTAGTGGTTGTGAAGGTTT | Reverse primer for verifying integrations at I1; primes 90bp downstream of I1R |
| pUDP002-F | **TCGGACGAGCTTACTCGTTTCGTCCTCACGGACTCATCAG**GTTTGTTTGTTTATGTGTGTTTATTC | Forward primer for pUCC001 backbone |
| pUDP002-R | **GCGCCGGCTGGGCAACATGCTTCGGCATGGCGAATGGGAC**CACAGGCCCCTTTTCCTTTG | Reverse primer for pUCC001 backbone |
| Bsa-R | TACACGCGTTTGTACAGAAAAAAAAGAAAAATTTGA | Reverse primer to check for correct assembly of gRNA expression vector; to be used with sense oligo containing the target sequence |
| URA3_diag_F | CGGCAACAACTTCATCCACAGTTC | Forward primer to amplify and sequence targeted site at the *URA3* locus |
| URA3_diag_R | GGAAAGAGCAGCTGCTCATAGAAGT | Reverse primer to amplify and sequence targeted site at the *URA3* locus |
| HIS3_diag_F | GCTGACTCACTTCTGTGATGATCATTAAAGC | Forward primer to amplify and sequence targeted site at the *HIS3* locus |
| HIS3_diag_R | TACTGGATAGACCGGAGTCGGAC | Reverse primer to amplify and sequence targeted site at the *HIS3* locus |
| ASR_J016CF | TCAAAGAATATCGTTGTCTTACCAG | Forward primer to amplify and sequence targeted site at the *LEU2* locus |
| ASR_J016CR | TGCCAAAATCTCCTTTACAGC | Reverse primer to amplify and sequence targeted site at the *LEU2* locus |
| Diag_KU80-F | CCGACGTTTGGTCCATTG | Forward primer to amplify and sequence targeted site at the *YKU80* locus |
| Diag_KU80-R | ACGTTCCAGGATGTGTCTAG | Reverse primer to amplify and sequence targeted site at the *YKU80* locus |
| Diag_DNL4-F | CTTGGTGCCCGTTGTTTCCA | Forward primer to amplify and sequence targeted site at the *DNL4* locus |
| Diag_DNL4-R | CTTCTAACAATGAGTGTTTCTTG | Reverse primer to amplify and sequence targeted site at the *DNL4* locus |
| Diag_NEJ1-F | AGTAACCTAAGCGCTAGATTCG | Forward primer to amplify and sequence targeted site at the *NEJ1* locus |
| Diag_NEJ1-R | GCTTCCATTTGGAAAACTCTCG | Reverse primer to amplify and sequence targeted site at the *NEJ1* locus |

**Table S4. Plasmids from the Yeast Toolkit used in this study**

| Plasmid name | YTK part type | Insert | E.coli marker |
| --- | --- | --- | --- |
| pYTK001 | n/a | Cloning vector | CamR |
| pYTK002 | 1 | Connector LS | CamR |
| pYTK009 | 2 | *ScTDH3pr* | CamR |
| pYTK010 | 2 | *ScCCW12pr* | CamR |
| pYTK011 | 2 | *ScPGK1pr* | CamR |
| pYTK013 | 2 | *ScTEF1pr* | CamR |
| pYTK030 | 2 | *ScGAL1pr* | CamR |
| pYTK033 | 3 | mVenus | CamR |
| pYTK053 | 4 | *ScADH1t* | CamR |
| pYTK054 | 4 | *ScPGK1t* | CamR |
| pYTK067 | 5 | ConR1 connector | CamR |
| pYTK077 | 6 | *kanMX*/kanamycinR | CamR |
| pYTK083 | 8 | AmpR-ColE1 | AmpR |

**Table S5.** gRNA targets used in this paper. The PAM is omitted.

| **Gene targeted** | **Sequence (5’ to 3’)** | **Remarks** |
| --- | --- | --- |
| *LAC4* | GACATCTCTTAGGACAAGTT |  |
| *YKU80* | AACCCTCTGATATCGATACC |  |
| *DNL4* | AACCATTTAAACTCTGGAGA | From Nambu-Nishida et al. (2017) |
| *NEJ1* | AAAGCCTCAACGAAAGAAGA |  |
| *URA3* | AGGTTCTTTCGTAACTTCCT |  |
| *HIS3* | ACCAACCAGAGTGCTTCGCT | Used for strains ASR.007 and ASR.024 |
| *HIS3* | CTTGATAATGCTTCGTCCAA | Used for strains ASR.008 and ASR.025 |
| *LEU2* | TTGAAACCTGAGTACGCCAA |  |

**Table S6.** Selected transcriptome data used for the selection of inducible promoters from Lertwattanasakul et al. (2015), alongside measured YFP values from this study.

| Gene/Promoter | Condition | Basal value | Induced value | Measured basal YFP | Measured induced YFP |
| --- | --- | --- | --- | --- | --- |
|  |  | (TSS-count in ppm, mean±s.d.) | (TSS-count in ppm, mean±s.d.) | (YFP/OD, mean±s.d.) | (YFP/OD, mean±s.d.) |
| *HSP150* | Heat*^a^* | 1762.1±753.5 | 3212.7±706.6 | 1097.6±210 | 1697.2±351.9 |
| *TSA1* | Heat | 829.2±152.3 | 1461.8±455.1 | 377.2±76 | 2359.8±286.6 |
| *HSP104* | Heat | 523±148.2 | 784.7±739.8 | 474.7±114.3 | 908.2±13.5 |
| *SSA2* | Heat | 150.5±37 | 539.3±467.9 | 429.5±231.7 | 1590±485.9 |
| *XYL1* | Xylose*^b^* | 106.4 | 250±0.04 | 70.5±46 | 753.7±83.2 |
| *XYL2* (annotated as sorbitol dehydrogenase) | Xylose | 536.3±297.6 | 1591.9±70.3 | 273±22.7 | 1722±155.3 |
| *ALD4* | Xylose | 2.5±0.9 | 581.5±144.9 | 145.5±39.7 | 1227.2±17.8 |
| *^a^* Transcriptome data was taken after 24h culture in YPD 45°C/30°C ,and YFP measurements after 24h culture in SD-ura with G418 at 42°C/30°C; *b* Transcriptome data was taken after 24h culture in YPX (yeast extract/peptone/2% xylose), and YFP measurements after 24h culture in SD-ura with G418 and 2% xylose as carbon source. | | | | | |

**Table S7.** Differences in promoter sequence identity between four *Kluyveromyces marxianus* strains. The promoter sequences from CBS6556 (or other strains) were compared against the same regions in three other commonly-used strains. Regions with differences were scanned for transcription factor binding site motifs in YeTFaSCo (De Boer and Hughes, 2012), using a curated set of binding site motifs (‘Expert Curated – no dubious’). A motif with at least 75% was set as a threshold for a positive match. Promoter sequences were taken from the sources cited in the main text. Promoters with 100% sequence identity were the sequences cloned in Table 2 and Table S1.

| Promoter | Sequence identity relative to CBS6556 | | | | Comments |
| --- | --- | --- | --- | --- | --- |
|  | NBRC1777 | DMKU3-1042 | CBS397 | CBS6556 |  |
| *PGK1* | 93% | 93% | 93% | 100% | Extra Gcr1 motifs in NBRC1777, DMKU3-1042 (1 each) and CBS397 (3) |
| *PDC1* | 92% | 92% | 98% | 100% |  |
| *ENO* | 99% | 99% | 96% | 100% |  |
| *TDH1* | 100% | 98% | 98.1/91.3%^a^ | 100% | Insertions in CBS397 core promoters |
| *HSP150* | 100% | 99% | 98.1/97.8% | 100% |  |
| *INU1* | 97% | 96% | 97% | 100% |  |
| *TEF1* | 96.9% | 97% | 98.3% | 100% |  |
| *REV1* | 98% | 91% | 88.4%/86.9% | 100% | Deletions (20-30bp) in repetitive region at -90 for DMKU3-1042 and CBS397 |
| *ALD4* | 100% | 99% | 94.4% | 100% | Deletions near Start codon in DMKU3-1042 |
| *GDH2* | 99.90% | 99.60% | 94.4% | 100% |  |
| *HHF1* | 89.80% | 88.90% | 92.50% | 100% |  |
| *TSA1* | 100% | 99.40% | 98.60% | 100% |  |
| *HSP104* | 96.70% | 96.50% | 91.60% | 100% |  |
| *SSA2* | 99.23% | 99.50% | 99.6%/99.60% | 100% |  |
| *TDH3* | 100% | 100% | 89% | 89% | Missing Gcr1 site in CBS6556 promoter |
| *FBA1* | 100% | 100% | 100% | 100% |  |
| *XYL1* | 97.60% | 98.90% | 92.60% | 100% |  |
| *XYL2* | 100% | 99.50% | 99.80% | 100% |  |
| *LAC4* | 95.60% | 95.60% | 100% | 95.70% |  |

^a^Differences for the two copies of the promoter in the diploid strain CBS397 (Varela et al., 2018).

**
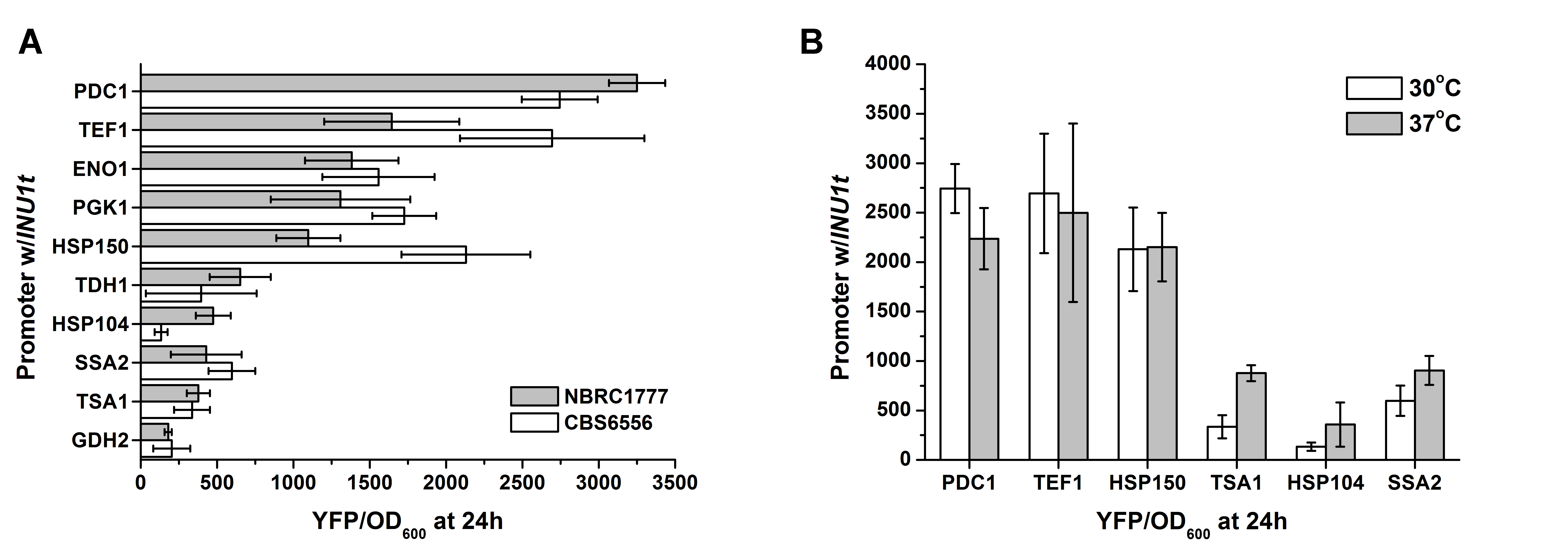
**

**Figure S1.** Characterization of promoters in *K. marxianus* CBS6556. (**A**) Comparative expression of selected promoters in strains NBRC1777 and CBS6556. (**B**) Characterization of heat-inducible promoters at 37°C. Induction at higher temperatures was not possible due to poor growth of CBS6556. All data are normalised to cell number using OD, and are plotted as the mean ± s.d. of at least three replicates.

**
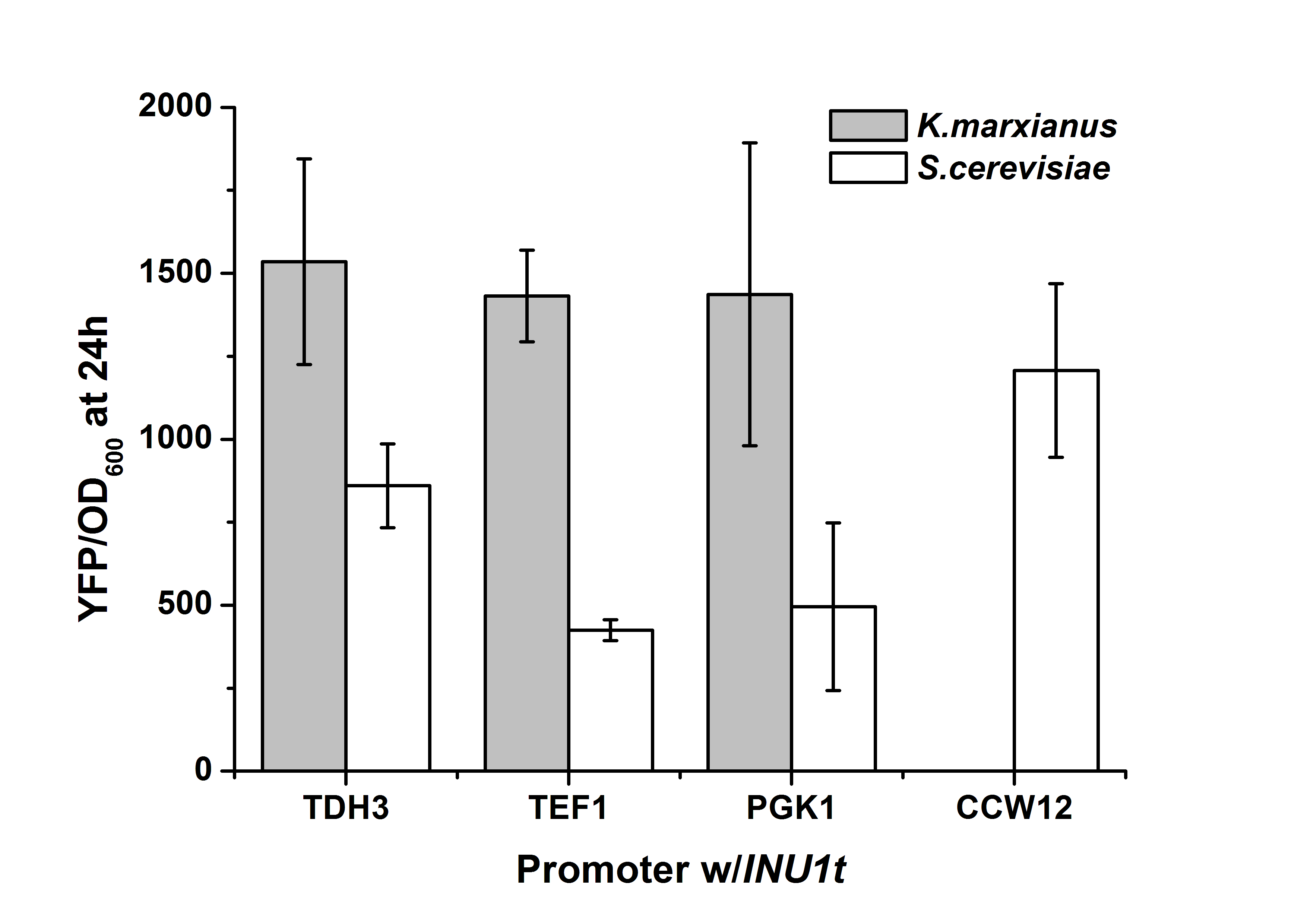
**

**Figure S2.** Expression behaviour of *S. cerevisiae* promoters in *K. marxianus*, alongside their *K. marxianus* orthologues. The promoters from the YTK were used in constructing the YFP expression vectors: YTK009 (*TDH3pr*), YTK010 (*CCW12pr*), YTK011 (*PGK1pr*) and YTK013 (*TEF1pr*). *CCW12* has no known orthologue in *K. marxianus*.

**
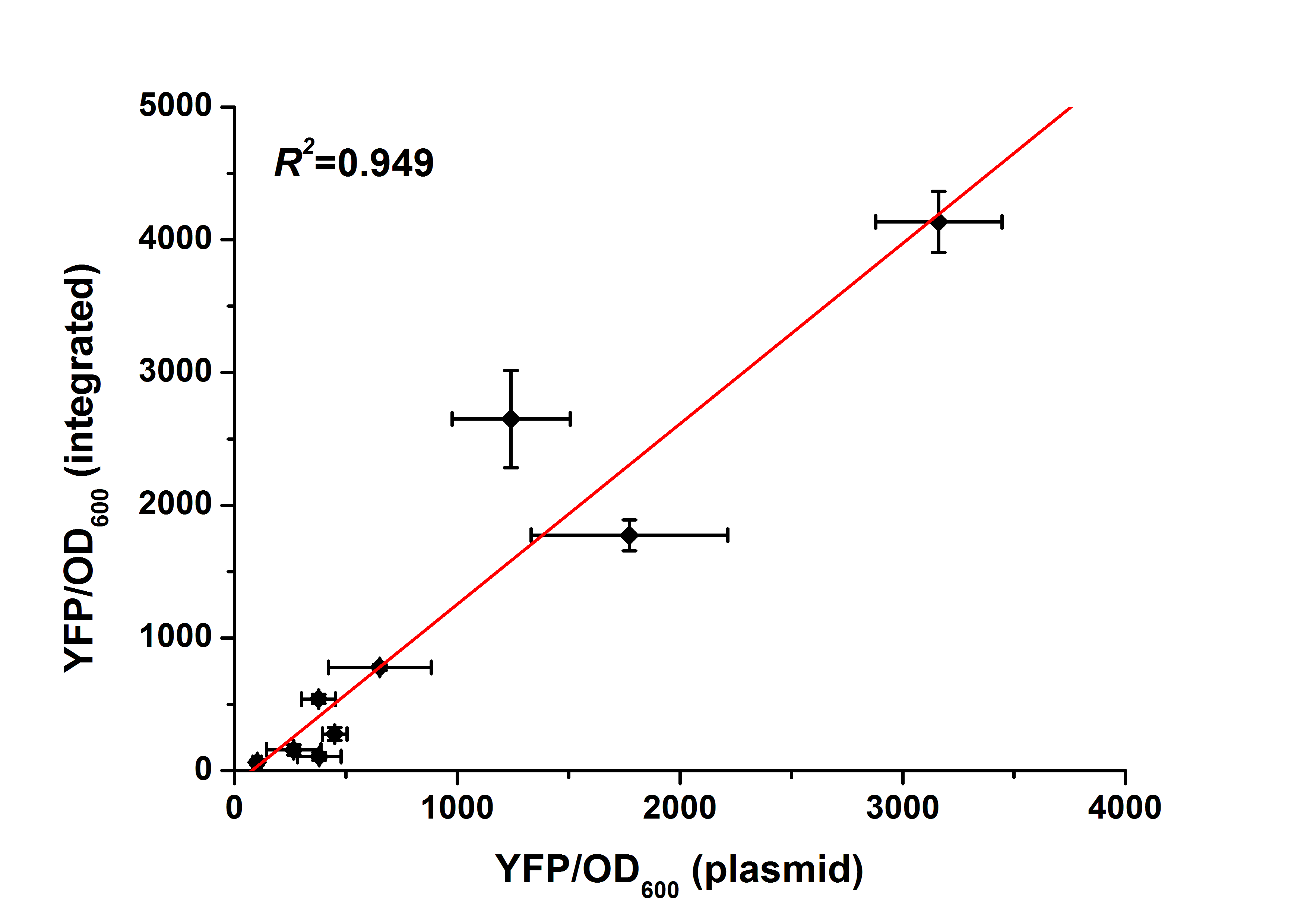
**

**Figure S3.** Correlation between chromosomal and plasmid-borne expression. YFP expression cassettes with selected constitutive promoters and *INU1t* were assembled into integrative vectors with G418 resistance and transformed into KmASR.005. G418-resistant colonies with the cassette integrated (as verified by colony PCR) into I1 (*LAC4*) were selected for fluorescence measurement on a plate reader. All data are normalised to cell number using OD, and are plotted as the mean ± s.d. of at least three replicates.

**Supplementary References**

De Boer, C. G., and Hughes, T. R. (2012). YeTFaSCo: A database of evaluated yeast transcription factor sequence specificities. *Nucleic Acids Res.* 40, 169–179. doi:10.1093/nar/gkr993.
